# Quantitative proteomics reveal lineage-specific protein profiles in iPSC-derived Marfan syndrome smooth muscle cells

**DOI:** 10.1038/s41598-020-77274-w

**Published:** 2020-11-23

**Authors:** Cristiana Iosef, Albert J. Pedroza, Jason Z. Cui, Alex R. Dalal, Mamoru Arakawa, Yasushi Tashima, Tiffany K. Koyano, Grayson Burdon, Samantha M. P. Churovich, Joshua O. Orrick, Mitchel Pariani, Michael P. Fischbein

**Affiliations:** 1 Department of Cardiothoracic Surgery, Stanford University, 300 Pasteur Dr, Falk CVRB, Stanford, CA 94305 USA; 2 Department of Pediatrics-Genetics, Stanford University, Stanford, CA USA

**Keywords:** Aneurysm, Aortic diseases, Induced pluripotent stem cells

## Abstract

Marfan syndrome (MFS) is a connective tissue disorder caused by mutations in the *FBN1* gene that produces wide disease phenotypic variability. The lack of ample genotype–phenotype correlation hinders translational study development aimed at improving disease prognosis. In response to this need, an induced pluripotent stem cell (iPSC) disease model has been used to test patient-specific cells by a proteomic approach. This model has the potential to risk stratify patients to make clinical decisions, including timing for surgical treatment. The regional propensity for aneurysm formation in MFS may be related to distinct smooth muscle cell (SMC) embryologic lineages. Thus, peripheral blood mononuclear cell (PBMC)-derived induced pluripotent stem cells (iPSC) were differentiated into lateral mesoderm (LM, aortic root) and neural crest (NC, ascending aorta/transverse arch) SMC lineages to model MFS aortic pathology. Isobaric Tags for Relative and Absolute Quantitation (iTRAQ) proteomic analysis by tandem mass spectrometry was applied to profile LM and NC iPSC SMCs from four MFS patients and two healthy controls. Analysis revealed 45 proteins with lineage-dependent expression in MFS patients, many of which were specific to diseased samples. Single protein-level data from both iPSC SMCs and primary MFS aortic root aneurysm tissue confirmed elevated integrin αV and reduced MRC2 in clinical disease specimens, validating the iPSC iTRAQ findings. Functionally, iPSC SMCs exhibited defective adhesion to a variety of extracellular matrix proteins, especially laminin-1 and fibronectin, suggesting altered cytoskeleton dynamics. This study defines the aortic embryologic origin-specific proteome in a validated iPSC SMC model to identify novel protein markers associated with MFS aneurysm phenotype. Translating iPSC findings into clinical aortic aneurysm tissue samples highlights the potential for iPSC-based methods to model MFS disease for mechanistic studies and therapeutic discovery in vitro.

## Introduction

Marfan syndrome (MFS) is an inherited connective tissue disorder (1/5,000 individuals) caused by a genetic mutation in the *FBN1* gene^1^. Patients typically develop aortic root aneurysms with ensuing aortic dissection and rupture remaining the leading cause of death^1–3^. Utilizing murine models, several investigators report this mutation results in pathologic enhanced transforming growth factor-β (TGF-β) signaling, although the downstream molecular events that lead to aneurysm development remain unknown^4,5^. However, uncertainty remains about the contribution of TGF-β signaling to early aneurysm development^6^. Moreover, whether this mechanism has fidelity with the human disease has not been firmly established. Currently, only prophylactic surgical replacement of the aortic root effectively increases life expectancy^7^. Medical care guidelines for elective surgery are based on aortic size or growth rate, without personalization to the individual patient.

Lineage studies have shown that vascular smooth muscle cells (SMC) in the different aortic anatomic segments have distinct embryologic origins, specifically, the aortic root is derived from the lateral mesoderm (LM) and the ascending aorta and arch are derived from the neural crest (NC)^8,9^. We hypothesize that site-specific development of aneurysms to the aortic root is predicated on these distinct SMC embryologic origins. Several investigators have generated human induced-pluripotent stem cell (iPSC)-derived vascular SMC subtypes from each embryologic origin to study vascular diseases, including aneurysm formation^10–13^. This unique model affords the opportunity to identify early pathologic mechanisms in aneurysm formation in vitro, in contrast to utilizing primary SMCs derived from aortic surgical specimens after aneurysms have already developed. Moreover, this strategy allows us to study an embryologic origin-specific SMC population, not contaminated with other aortic wall resident cells (fibroblasts, inflammatory cells, endothelial cells, or SMCs derived from a different embryologic origin). Sinha et al. initially reported origin-specific SMCs demonstrated differential matrix metalloproteinase (MMP) activation in response to IL-1β^10^. Subsequently, utilizing an MFS iPSC-derived SMC model, Granata et al. concluded that TGF-β signaling through the non-canonical pathway may be protective or detrimental depending on the stage of neural crest SMC maturity^11^. Importantly, these findings were performed in iPSC-derived NC SMCs, although the LM is the embryologic origin where aneurysms develop in MFS.

Herein, we used a peripheral blood mononuclear cells (PBMC)-derived iPSC model system to generate iPSCs from MFS patients^14–20^. To enhance our current knowledge of lineage-specific SMC contribution to aneurysm formation, we performed Isobaric Tags for Relative and Absolute Quantification (iTRAQ) proteomic analysis on iPSC-derived LM and NC SMCs from four MFS and two control patients. By directly studying proteomic differences in LM vs. NC SMCs within the same patient, we highlight the role embryologic origin plays on processes occurring focally in aortic root during aneurysm formation. Moreover, we further validate the iPSC system as a faithful model for MFS pathology, underscoring its potential as an “aorta in a dish” system to predict clinical severity by molecular phenotyping rather than relying on serial radiographic imaging and generalized knowledge of FBN1 mutation subtypes. This model system opens the door for future use of patient-derived iPSC in precision medicine strategies to tailor treatments for individual patients^21,22^.

## Results

### iPSC SMC derivation to model MFS aortic pathology

**Figure 1:**
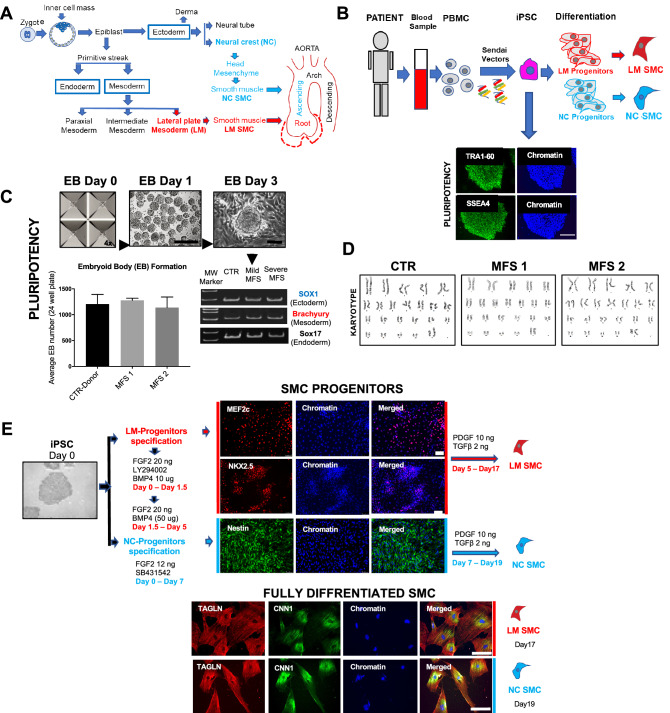
Peripheral blood mononuclear cells (PBMC) derived iPSCs differentiated into vascular smooth muscle cells (SMC) can model Marfan syndrome disease, in vitro. (**A**) Brief description, lineages contributing SMCs to the thoracic aorta. While lateral mesoderm (LM) SMCs contribute to the aortic root structure, neural crest (NC) SMCs populate the ascending aorta. (**B**) PBMC reprogramming with Sendai virus vectors of OCT4, SOX2, Nanog and cMyc transcription factors to generate induced pluripotent stem cells (iPSC) which were further differentiated into LM and NC SMC progenitor cells. IPSCs stained positively for pluripotency markers TRA1-60 and SSEA4. (**C**) Embryoid body (EB) formation assay with representative photos of the AgreWell cell funneling system (day 0), embryoid bodies (spherical aggregates) at day 1 post culture and a representative EB with spontaneous differentiation at day 3. Bar graph shows the total number of spherical aggregates per iPSC line, formed in one well of a 24-well plate. Gel images demonstrate PCR results from EB for SOX-1 marker for ectoderm, Brachyury for mesoderm and SOX-17 for endoderm. (**D**) Representative karyotype (G-banding) for each iPSC line. (**E**) LM progenitor cells (MEF2C- and NKX2.5-positive) were generated from iPSC using AKT/PKB inhibitor (LY294002) and FGF2, then FGF2 and BMP4. NC progenitor cells (Nestin-positive) were generated from iPSC using an Activin/TGF-β1 inhibitor (SB431542) and FGF2 for 7 days. Cells were further differentiated into SMCs using PDGF-BB and TGF-β1 in a 12-day protocol. Resulting cells presented SMC phenotype, positive for transgelin (TAGLN) and calponin 1 (CNN1).

To model embryologic origin-specific susceptibility to causative *FBN1* mutations, iPSCs from each MFS patient and healthy controls were differentiated through LM and NC progenitor stages followed by parallel SMC differentiation using a validated protocol (Fig. 1A)^23^. *FBN1* mutations were verified before and after iPSC derivation by DNA sequencing. Prior to differentiation, pluripotency was confirmed via the following: (a) positive staining for stage specific embryonic antigen 4 (SSEA4) and tumor receptor antigen 1–60 (TRA1-60) embryonic markers (Fig. 1B); (b) embryoid body (EB) formation of optimal density; and (c) spontaneous differentiation of EBs after 72 h with expression of specific markers from each of the three embryonic layers (ectoderm/SOX-1, mesoderm/brachyury, and endoderm/SOX-17) (Fig. 1C). Karyotyping following iPSC induction confirmed the absence of major chromosomal changes (translocations, deletions or crossover events) as a result of reprogramming (Fig. 1D).

iPSCs were then differentiated into NC and LM progenitors. For NC differentiation, cells were grown in a chemically defined medium (CDM) formula adjusted with fibroblast growth factor 2 (FGF2) and TGF-β inhibitor SB431542 for 7 days. Nestin, a marker of NC lineage^24^, was detectable by immunocytochemistry/immunofluorescence (ICC/IF) at this stage (Fig. 1E). For early mesoderm formation, cells were initially grown in CDM adjusted with FGF2 and supplemented with AKT/PKB inhibitor (LY294002) for 36 h. Further specification into LM progenitors required CDM containing FGF2 and adjusted with bone morpho-genetic protein 4 (BMP4) for an additional 3.5 days. ICC detection of nuclear myocyte-specific enhancer factor 2C (MEF2C) and NK2 Homeobox 5 (NKX2.5) at this stage confirmed LM transition to secondary heart field identity^25^. Both NC- and LM-progenitor cells were further differentiated into iPSC derived SMCs using a CDM formula with platelet-derived growth factor (PDGF) and TGF-β1 for 12 days, when both LM and NC SMCs stained positively for SMC contractile proteins smooth muscle actin (SMA), transgelin (TAGLN) and calponin 1 (CNN1).

### iTRAQ analysis produced a robust proteomic spectrum of the iPSC SMC

While high throughput technologies such as RNA sequencing may decipher pathological mechanisms^26^, the proteomic profile represents the ultimate signature of cellular function represented by the final protein products downstream of gene transcription. Therefore, we applied iTRAQ tandem spectrometry to investigate MFS pathogenesis using the iPSC SMC model. Protein extracts from iPSC SMC lines derived through the LM and NC pathways from both MFS and control lines were utilized and labeled with isotope-encoded reporter ions according to manufacturer protocols.

**Figure 2:**
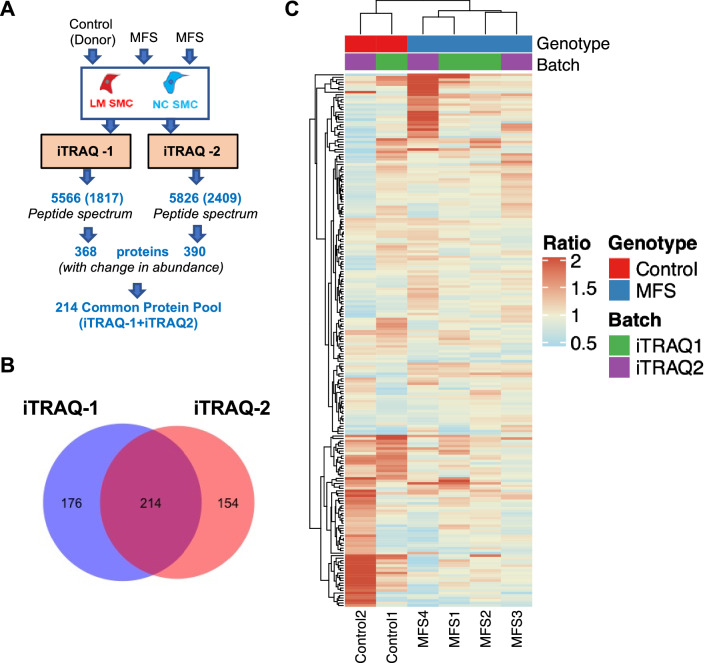
Bioinformatic analysis reveals distinct lineage-specific proteomic profile of Marfan syndrome (MFS) iPSC SMCs compared to donor control. (**A**) Technical description of two independent iTRAQ experiments (workflow and data paring). (**B)** Data merging between two iTRAQ runs resulting in “common” protein pool of 214 proteins represented in both experiments selected for further analysis. (**C**) Hierarchical clustering of iPSC SMC iTRAQ data from MFS patients (n = 4) and controls (n = 2) for common 214-protein pool. Each sample analyzed for ratio of expression between LM and NC lines from the individual patient. Samples cluster primarily by disease vs. control, rather than iTRAQ batch.

Two 8-plex LC–MS/MS shotgun tests (labeled iTRAQ-1 and iTRAQ-2) produced spectra of 5566 and 5826 accountable peptides, respectively. After removal of the low-scoring peptides [cut-off ion score was calculated at a confidence interval of > 95%], 1817 peptides accounting for 32.64% of the iTRAQ-1 original pool (5566) and 2409 peptides accounting for 41.34% of the iTRAQ-2 original pool (5826) were selected based on their change in abundance. The iTRAQ-1 1817 peptide spectrum corresponded to 368 proteins and the iTRAQ-2 2409 peptide spectrum corresponded to 390 proteins. (Fig. 2A, Supplemental Table 1 and 2). The iTRAQ-1 protein pool (368) demonstrated heterogeneous molecular weights, as follows: 22.07% (20–40 kDal), 32.98% (40–80 kDal), 23.11% (80–160 kDal), and 21.55% (> 160 kDal). Additionally, 4 markers out of the total of 368 pool had peptide coverage between 40 to 50%, 9 (30–40%), 25 (20–30%), 77 (10–20%) and the rest of the 253 markers were covered from 1 to 10%. Molecular weights for the iTRAQ-2 (390) protein pool were as follows: 32.3% (20–40 kDal), 35.5% (40–80 kDal), 10.5% (80–100 kDal) and 18.3% (> 160 kDal). The summary of the peptide coverage for the iTRAQ-2 experiment included: 11 (40–50%), 26 (30–40%), 40 (20–30%), 77 (10–20%), and 231(1–10%) (Supplemental Table 1 and 2). To permit composite analysis of the two runs, a common protein pool consisting of 214 proteins represented in both iTRAQ datasets was identified and used in downstream analysis (Fig. 2B).

### Marfan iPSC SMCs from different embryologic origins have unique proteomic patterns

**Figure 3:**
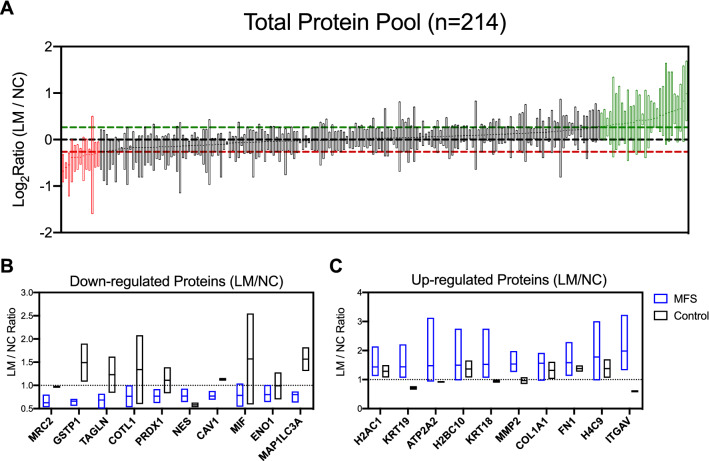
Single protein-level data. (**A**) Proteins with change in abundance per iTRAQ analysis. Protein expression ratios (LM/NC) for all 214 “common” proteins between iTRAQ runs displayed. Proteins with mean increase expression > 1.2 in LM (green line) highlighted in green, and increased expression in NC > 1.2 (red line) highlighted in red. Boxes depict range for n = 4 MFS samples, line depicts mean. (**B**) Top 10 most highly downregulated in LM SMC from MFS patients. (**C**) Top 10 most highly upregulated in LM SMC from MFS patients. Blue boxes depict range for MFS patients (n = 4), black boxes depict range for controls (n = 2). Lines depict mean.

Despite systemic effects of *FBN1* mutations, patients with MFS typically develop aortic root aneurysms. To test our hypothesis that lineage-specific changes affecting SMCs populating the aortic root via the LM development pathway contribute to this phenomenon, proteomic profiles were compared between MFS LM and NC SMCs. We analyzed each individual patient sample independently by determining the LM/NC expression ratio for each protein to minimize batch effect induced by multiple dataset integration. Hierarchical clustering of individual patient proteomic data revealed distinct profiles for control (n = 2) and MFS patients (n = 4) independent of iTRAQ batch, suggesting disease-specific protein expression patterns (Fig. 2C). We next ranked individual proteins by mean expression ratio in MFS patients to determine which proteins may exhibit disease-specific patterns. Using a cutoff of 1.2-fold up- or down-regulation, we identified 13 proteins with reduced expression and 43 proteins with increased expression in MFS LM compared to NC SMCs (Fig. 3A). Among proteins down-regulated in MFS LM SMCs, mannose receptor C type 2 (MRC2) was reduced in all MFS lines (mean ratio to NC 0.62, range 0.53–0.80) while control lines exhibited uniform expression between lineages (0.95–0.99 ratio to NC) (Fig. 3B). Transgelin (TAGLN), a classic marker for SMC differentiation was also reduced in MFS LM SMC samples (mean ratio to NC 0.68, range 0.43–0.82) suggesting relatively impaired contractile phenotype in the LM lineage, a feature not seen in control lines (ratio 0.84–1.62). Interestingly, reduced expression of the autophagy-related microtubule-associated proteins 1A/1B light chain 3A (MAP1LC3A) was consistently observed in MFS LM SMC lines (mean ratio 0.80, range 0.63–0.84). Nestin (NES), a NC lineage marker, was consistently downregulated in all LM lines regardless of disease phenotype (mean ratio 0.72, range 0.53–0.93).

We next analyzed proteins with enriched expression in the MFS LM SMC samples. Most strikingly, integrin αV (ITGAV) was upregulated in all four MFS LM SMC lines (mean ratio to NC 1.98, range 1.32–3.23) while reduced in control lines (mean 0.59, range 0.56–0.63), suggesting disease-specific activation of this receptor in the MFS LM SMCs. Similarly, matrix metalloproteinase-2 (MMP2), a known contributor to ECM proteolysis in MFS aortic aneurysm development in MFS^27–31^ was increased in all MFS LM SMC lines (mean ratio 1.53, range 1.26–1.98), while MMP2 was stably expressed in control LM and NC lineages (mean ratio 0.96, range 0.85–1.08). LM SMCs from MFS and control patients showed increased expression of multiple critical aortic ECM proteins including collagen type 1, alpha 1 (COL1A1, mean ratio to NC 1.48, range 0.96–1.92) and fibronectin (FN1, mean 1.52, range 1.13–2.29) (Fig. 3C). Expression of laminin subunit gamma-1 (LAMC1) was consistently enriched in MFS LM SMCs (mean ratio 1.37, range 1.20–1.73) with variable expression in controls (mean ratio to NC 1.02, range 0.51–1.52). These data indicate that lineage-specific protein expression may contribute to heterogeneous regional aortic ECM substrate.

### iTRAQ identifies MFS disease-specific protein expression in iPSC model

**Figure 4:**
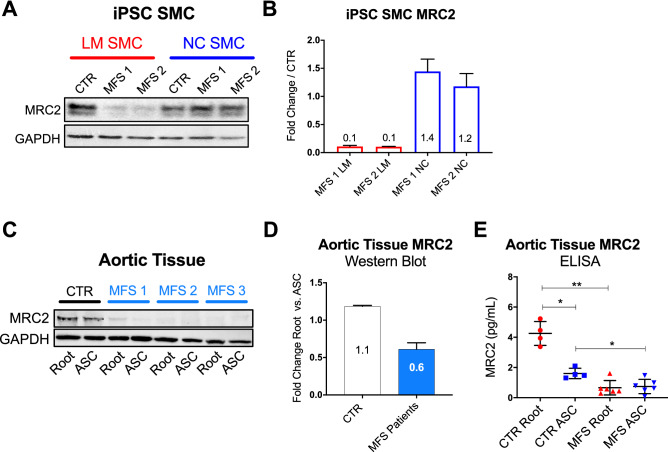
Downregulated MRC2 expression predicted by iPSC SMC iTRAQ analysis is dysregulated in human MFS aortic aneurysm specimens. (**A**) Western blot analysis of mannose receptor 2 (MRC2) in both LM and NC originated iPSC SMC; (**B**) Quantification of MRC2 expression ratio for MFS patients to control in denoted lineage. (**C**) Representative western blot analysis of mannose receptor 2 (MRC2) in human aortic tissue lysates from MFS patients (n = 3) and healthy organ donor control. Root = aortic root (Sinus of Valsalva), ASC = ascending aorta. (**D**) Quantitative comparison of Root vs ASC MRC2 expression by western blot in aortic tissue samples. (**E**) ELISA results of MRC2 levels in aortic tissues of control patients (N = 4) vs. MFS patients (N = 6). Western blots from iPSC SMC lines represent at least three technical replicates from the indicated sample. Groups tested by ELISA were compared by non-parametric analysis, Mann–Whitney rank sum test. Significance is assigned based on the *P*-value (*P* < 0.05, **P < 0.01, ***P < 0.001) where n stands for biological replicates (n = 4 for controls, and n = 6 for MSF samples).

While comparing LM to NC SMC protein ratios within individual patients permits the integration of multiple iTRAQ datasets, this approach complicates head-to-head comparisons of expression levels between MFS patients and controls. To further investigate proteins of interest from the iTRAQ data, we performed western blots on iPSC derived LM and NC SMCs. MFS iPSC SMCs showed uniquely down-regulated MRC2 expression in the LM lineage. Because down-regulation of MRC2, a cell surface receptor responsible for internalization and degradation of extracellular collagen, is predicted to contribute to aberrant vesicle-mediated trafficking of ECM components, this protein represents a novel potential contributor to ECM derangement in MFS^32–34^. Western blot confirmed LM-specific reduction of MRC2 expression in MFS patients compared to healthy control (Fig. 4A–B). To determine whether reduced MRC2 is a feature of clinical MFS aneurysm, we assessed MRC2 expression via western blot from tissue lysates obtained from MFS patients undergoing aneurysm repair surgery. Reduced MRC2 was confirmed in human MFS aortic tissue lysates from patients with aortic root aneurysms (n = 3) compared to a healthy organ donor control (MRC2 aortic root/ascending: MFS 0.6 vs. control 1.1-fold, Fig. 4C–D). For quantitative confirmation using a larger sample pool, enzymatic linked immunosorbent assay (ELISA) was performed comparing healthy controls (n = 4) to MFS patients (n = 6). MRC2 levels were significantly reduced in both MFS aortic root and ascending tissue compared to control-matched specimens (MFS root/control root: 0.12-fold, P = 0.0095; MFS ascending/control ascending: 0.467-fold, P = 0.038) (Fig. 4E). Collectively these data identify MRC2 down-regulation as a novel marker of MFS aortic aneurysm identified in vitro by shotgun proteomic techniques.

**Figure 5:**
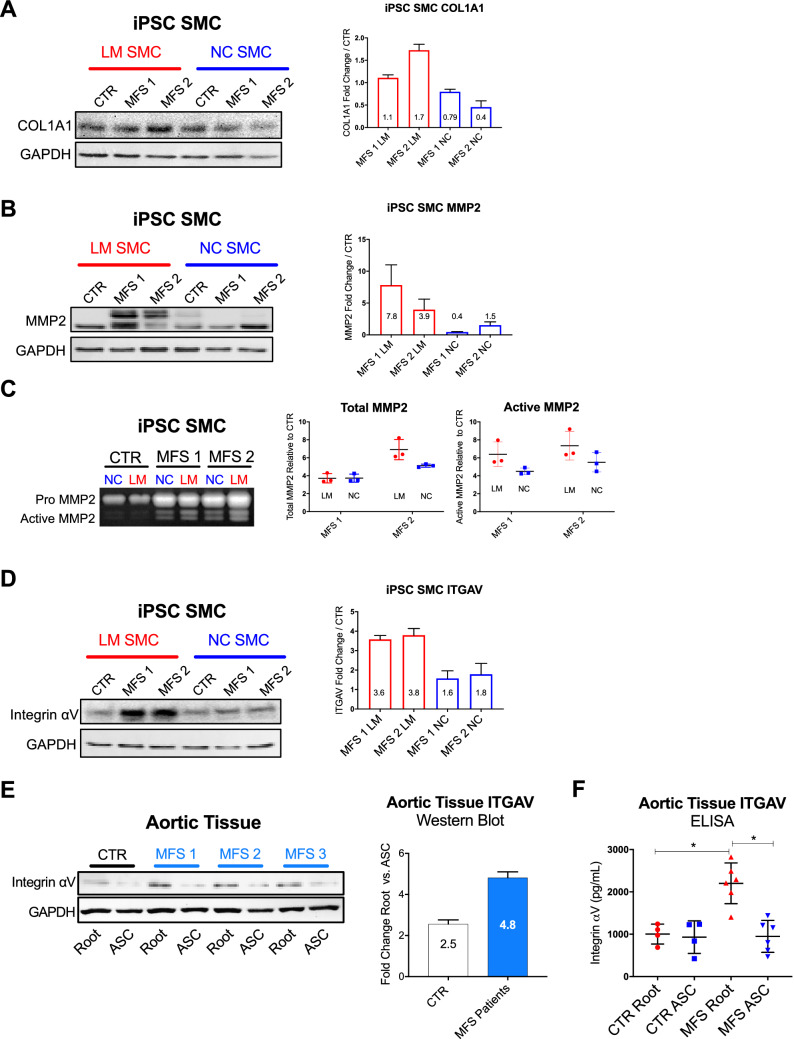
ITGAV, COL1A1, and MMP2 expression are increased in MFS iPSC SMCs. Western blot results for (**A)** COL1A1 and (**B**) MMP2 expression in iPSC SMCs. Expression is increased in MFS LM samples compared to control. (**C**) MMP2 gelatin zymography representative gel image and quantitation. (**D**) ITGAV western blot in iPSC SMC system shows pronounced ITGAV increase in MFS LM compared to control. (**E**) ITGAV western blot in human aortic tissue lysates from MFS patients (n = 3) and healthy control showing increased expression in the aneurysmal aortic root segment in MFS patients. (**F**) Aortic tissue protein lysate ELISA results comparing MFS patients (n = 6) to healthy controls (n = 4). Western blots and MMP assay from iPSC SMC lines represent at least three technical replicates from the indicated sample Groups tested by ELISA were compared by non-parametric analysis, Mann–Whitney rank sum test. Significance is assigned based on the *P*-value (*P* < 0.05, **P < 0.01, ***P < 0.001).

We next assessed COL1A1 and MMP2 expression in the iPSC SMC system. Enriched expression of these proteins has been identified in both murine models of MFS and human MFS aortic tissue samples^35,36^. In addition to the enriched LM:NC ratio identified by iTRAQ, western blot confirmed MFS patient lines overexpressed COL1A1 and MMP2 relative to the control line (Fig. 5A–B). Furthermore, gelatin zymography demonstrated an increase in MMP2 activity in MFS iPSC SMCs, with enhanced activity in LM lineage relative to NC (Fig. 5C). These results confirm that the iPSC model faithfully recapitulates classic markers of MFS aortic aneurysm pathogenesis^26,30,31,36^.

We next confirmed ITGAV overexpression in MFS LM SMCs. Consistent with iTRAQ results, ITGAV was highly enriched in MFS LM SMC samples compared to either control or paired NC samples (Fig. 5D). ITGAV protein levels were also increased in aortic root aneurysm tissue from MFS patients compared to normal control (MFS 4.8 vs. control 2.5-fold aortic root/ascending, Fig. 5E). Quantitative ELISA using human aortic root and ascending aortic tissues (MFS patients: n = 6; control patients: n = 4) confirmed significantly higher ITGAV in MFS aortic root tissue compared to either control roots (MFS root/control root: 2.1-fold, P = 0.0095) or to MFS case-matched non-dilated ascending aortic tissue (MFS root/MFS ascending: 2.3-fold, P = 0.043, Fig. 5F).

### iPSC SMCs from MFS patients display reduced adhesion to common ECM substrates

**Figure 6:**
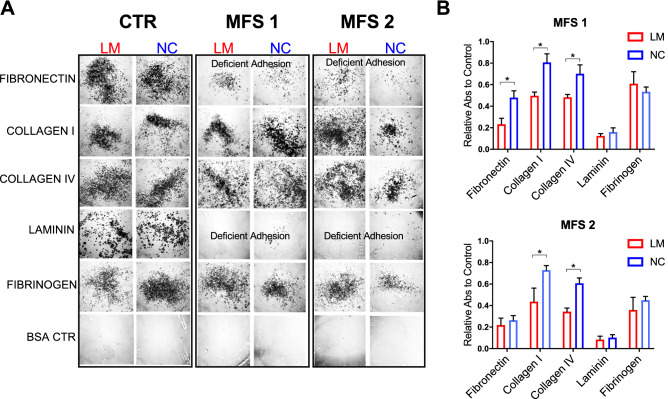
iPSC SMCs from MFS patients have cell adhesion deficiencies, in vitro. Cell staining and quantification of cell adhesion assay. Quantification results depict absorbance values for MFS iPSC SMCs normalized to donor control levels and plotted as average of two different experiments with three technical replicates. Normalized LM SMC absorbance was compared to NC SMCs. Groups were compared by non-parametric analysis (Mann–Whitney rank sum test). *P < 0.05.

Because iTRAQ analysis identified disease-specific protein expression of multiple ECM components and ITGAV, a surface receptor with adhesion and signaling functions, we hypothesized that MFS iPSC SMCs may display altered adhesive properties^37–39^. We tested and quantitated LM and NC SMC adhesion to various matrix compositions using a standard Cell Adhesion Assay based on colorimetric assessment. Cells were plated in wells pre-coated with fibronectin, collagen I or IV, laminin, fibrinogen or bovine serum albumin (BSA) as negative control. MFS SMCs from both LM and NC lineages displayed reduced adhesion to all tested substrates compared to control SMCs (Fig. 6A–B). Although collagen (I, IV) and fibrinogen binding was reduced compared to control, SMCs derived from both MFS patients had markedly poor adhesion to fibronectin and laminin. Moreover, when comparing MFS LM vs. NC SMCs, LM SMCs seemed to have reduced binding.

## Discussion

Although MFS is a systemic disease, the pathophysiology leading to localized aortic root aneurysm formation remains unresolved. While hemodynamic and other biomechanical factors may play a role, we hypothesize that distinct embryologic origins of thoracic aortic segments result in variable regional aortic wall remodeling. We have developed iPSCs from patients with MFS and successfully differentiated them into SMC from different embryologic origins. Applying an iTRAQ proteomic strategy, iPSC derived SMCs from MFS patients are utilized to model and investigate human disease mechanisms and discover novel biological pathways that participate in early ECM degeneration.

**Figure 7:**
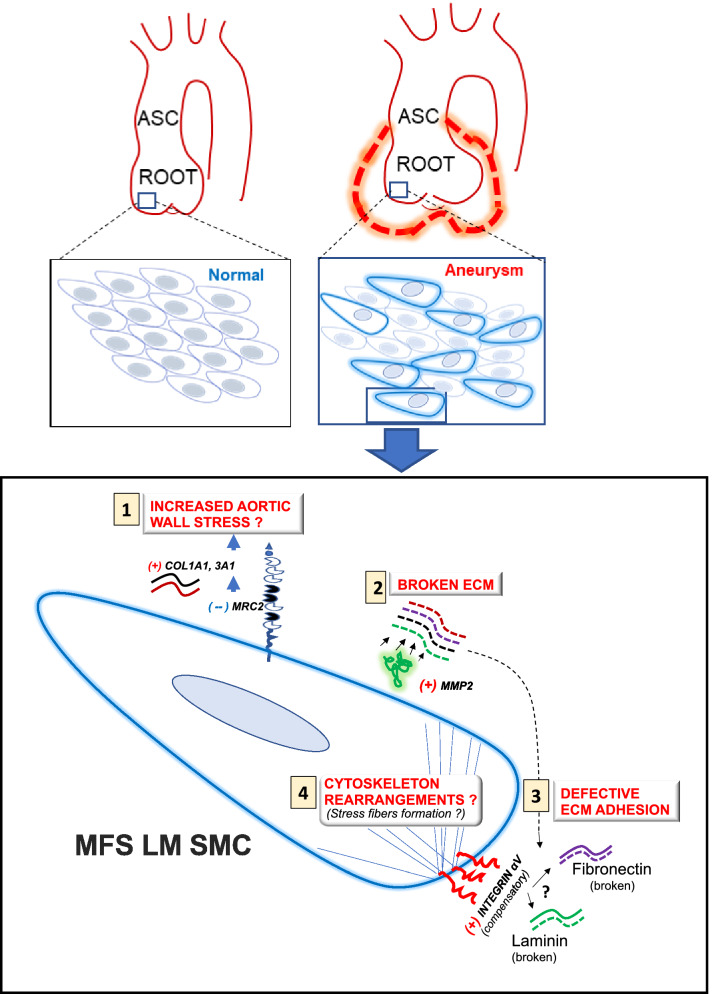
Proposed SMC lineage specific events during MFS aortic root aneurysm formation. (**1**) Reduced SMC mannose receptor 2 (MRC2) expression leads to decreased collagen internalization/breakdown, accounting for increased aortic collagen and wall stiffness. (**2**) Augmented matrix metalloproteinase-2 (MMP2) activity levels break down the extracellular matrix (ECM), which also may play a role in (**3**) defective ECM adhesion. Decreased SMC binding to the ECM, including laminin and fibronectin, causes a hypothetical reflexive increase in cell surface integrin αV expression. (**4**) LM SMCs in the aortic root have alterations within the cytoskeleton.

SMC modulation from contractile to the dedifferentiated, synthetic phenotype is known to contribute to aortic aneurysm development^3,35,40^. We recently reported on the “mixed SMC phenotype” in human MFS aortic tissue and primary vascular SMC lines, which overexpressed both contractile and synthetic genes^35^. Similarly, Crosas-Molist et al.^36^ observed an overexpression of both contractile markers and collagen I in human MFS specimens. Conversely, single cell transcriptomics in *Fbn1*^*C1041G*/+^ MFS mice revealed a dynamic cell state change to a phenotypically modulated state with reduced SMC contractile gene expression and enriched synthetic genes (fibronectin, collagens, and matrix metalloproteinases)^26^. While the iPSC-derived SMC model represents newly-formed, immature SMCs rather than “de-differentiated” cells identified in aneurysm tissue, we identified reduced expression of the contractile marker TAGLN in MFS LM compared to NC SMCs. Concurrently, increased “synthetic” proteins (MMP2, COL1A1) compared to normal control in the iPSC SMC model is completely consistent with human and mouse MFS reports, validating this platform as a tool for targeted cell-based assays aimed toward future drug/therapeutic discovery (Fig. 7).

Interestingly, we identified reduced MRC2 protein expression in MFS iPSC-derived SMCs. This novel finding was subsequently confirmed with western blot and ELISA in clinical MFS surgical aortic specimens. To the best of our knowledge, MRC2 has not been previously studied in aortic aneurysms. We postulate that MRC2 plays a key role in ECM remodeling by mediating the internalization and intracellular degradation of extracellular collagen^32,34,41^. Embryonic fibroblasts transfected with mutated MRC2 gene display impaired collagen binding and internalization, promoting an impaired migratory phenotype^33^. Moreover, MRC2 participates in extracellular collagen remodeling via membrane type-1 MMP (MT1-MMP) regulation in prostate cancer^42^. Reduced MRC2 protein expression is a particularly intriguing target for further study in MFS in the setting of simultaneously increased collagen expression. Whether collagen deposition accelerates aneurysm formation or represents a beneficial response to reinforce the aortic wall remains controversial. Collagen may increase aortic stiffness and predisposition to aneurysm and dissection. Corroborating this theory, Baumgartner et al. reported that high aortic wall distensibility was a favorable prognostic indicator, whereas reduced elasticity was predictive of aortic wall abnormalities in MFS patients ^43,44^.

Lineage-specific LM and NC SMCs intermingle in the aortic root, whereas they are spatially more distinct in the ascending aorta^8^. Therefore, we believe the complex interactions between aortic root MFS LM and NC SMCs, each with uniquely perturbed biological functions, participate in regional aneurysm formation. Supporting this hypothesis, MacFarlane et al. recently reported that aortic root aneurysm predisposition in murine Loeys-Dietz syndrome is dependent on the paracrine effects between defective Smad signaling in secondary heart field-derived SMCs and excessive Smad signaling in neighboring NC-derived SMCs^45^. Hypothesizing that *FBN1* mutations similarly mediate distinct effects on SMCs depending on aortic wall embryologic origin, we sought to characterize the protein expression patterns in LM vs. NC derived SMCs. Highly up-regulated proteins in MFS LM SMCs included ITGAV, COL1A1, MMP-2, FN and LAMC1. An important question remains how altered ECM composition and defects in SMC adhesion participate in MFS aneurysm development. Given that these proteins were strongly up-regulated in LM SMCs and subsequently validated in separate human MFS aortic root aneurysms, our initial theory was that each may contribute to focal aneurysm development. Integrins play an important role in cytoskeleton polymer assembly, cell signaling and cytoskeleton attachment to the ECM^37,38,46^. Consistent with our proteomic findings, Parker et al. analyzed the ascending aortic proteome within the MFS Fbn1^C1041G/+^ mouse model and noted an up-regulation in integrin αVβ3 expression^47^. Utilizing both in vivo and in vitro studies, they proposed that integrin overexpression enhances non-canonical TGF-β activation of rapamycin-independent component of mammalian target of rapamycin (Rictor), leading to modified SMC proliferation, migration and mitochondrial metabolism. Although a fascinating target for future investigation, because the functional adhesion assay revealed that MFS SMCs had poor adherence to fibronectin and laminin, both ligands for integrin αV^38^, increased MFS SMC integrin αV may not represent the initiating event, but instead a compensatory response to defective ECM adhesion. Despite this undefined pathologic role, ITGAV represents a promising candidate protein marker for disease progression in MFS.

Proteomic analyses have been used previously to investigate MFS aortic aneurysm pathology in a number of contexts. Using redox proteomics in human MFS aortic tissue, Jiménez-Altayó identified increased redox stress within multiple cytoskeletal proteins including ACTA2. Interestingly, enhanced oxidative stress was correlated with NADPH oxidase 4 (NOX4) expression. Nox4 expression was pathological in the *Fbn1*^*C1041G/*+^ model highlighting the importance of posttranslational modifications^48^. Pilop et al. demonstrated enriched calpain-mediated proteolysis resulting in increased C-terminal filamin fragments in both MFS and bicuspid aortic valve aortopathy as well as increased vinculin, calponin, and microfibril-associated glycoprotein-4 (MFAP4), further highlighting modulation of SMC function and ECM components as a central process in MFS pathophysiology^49^. MFAP4 over-expression and glycosylation was also identified by Yin et al. using glycoproteomic analysis, which was associated with ECM content modulation and ADAMTS protease expression. Cikach et al. further highlighted the collective involvement of these processes using ‘proteoglycanome’-specific methodology, reporting enhanced proteoglycan (aggrecan and versican) accumulation within the aortic tunica media of human and mouse MFS, which was theorized to disrupt ECM integrity and biomechanics. These studies, employing proteomic approaches to study human and mouse MFS aneurysm tissue, highlight significant ECM content modulation in MFS aneurysm.

Uniquely, we utilized proteomics in iPSC-derived SMCs to complement knowledge gained from animal models and human surgical specimens. This system allows us to study the lineage-specific role in aneurysm formation in vitro using SMCs from clinical patients with FBN1 mutations, providing a platform for novel molecular pathway discovery and potentially drug/therapeutic testing. Herein, our proteomic findings support the transcriptomic data identified by Cheung et al.^10^ who detected enriched clusters in MFS iPSC SMCs, including actin cytoskeleton binding and organization, ECM and receptor binding, calcium ion binding and cell–cell/cell-substrate adhesion. Importantly, their experiments were performed almost exclusively on NC SMCs while our analysis compares LM vs. NC SMC phenotypes. We demonstrated increased MMP and COL1A1 protein expression in LM compared to NC MFS iPSC SMCs. MFS aneurysms form specifically in the aortic root, sparing the ascending aorta and arch. Therefore, we believe studying the differences between LM (aortic root) and NC (ascending aorta/arch) SMCs are essential to model each aortic region, albeit mixing of both lineages likely occur within the root.

This study has multiple limitations. By studying individual patients using LM/NC ratios, we minimized batch effect across two iTRAQ runs and identified lineage-specific changes within single patients while potentially obfuscating proteomic differences between MFS and control lines (e.g. MFS LM vs control LM). Given the high degree of biologic variability between individuals, CRISPR-mediated correction of *FBN1* mutations from MFS patients represents an alternative strategy to confirm our findings are attributable to MFS aortopathy specifically. This analysis is also limited by the inclusion of only four MFS patients; nevertheless, we identified highly consistent findings for multiple proteins in all patients. In a larger study, a targeted protein assay for candidate biomarkers could be screened to identify protein expression signatures that correlate with clinical disease severity. Over 3000 *FBN1* mutations have been reported, from missense mutations to premature termination codons. MFS displays large variability in clinical severity with unpredictable correlation with the genetic mutation^50–52^. Classifying *FBN1* mutations as either dominant negative (incorporation of mutated with non-mutated fibrillin-1, leading to abnormal protein in the ECM) or haploinsufficient (non-mutated fibrillin-1 only, resulting in reduced fibrillin-1 protein in the ECM), Franken et al.^53,54^ reported that haploinsufficient patients had increased aortic root growth rate, dissection risk and cardiovascular death. Anecdotally, this study included one clinically severe MFS patient with a haploinsufficient phenotype, while three patients with dominant negative mutations displayed mild (2) and severe (1) clinical presentations (detailed genetics listed in Methods).

There is ongoing debate regarding the optimal timing for prophylactic surgery in MFS and other inherited aortopathies (Loeys-Dietz syndrome, bicuspid aortic valve)^55,56^. Currently, guidelines for prophylactic surgery are based on aortic size, family history and symptoms. Patients may present with life-threatening dissections at small aortic dimensions, whereas other patients may be operated on too early. In the future, the iPSC model could conceivably be deployed to identify molecular phenotypes in vitro as an adjunct, precision medicine-based risk stratification measure. Finally, the consistent and origin-specific protein expression changes we have identified in MFS iPSC SMCs support the promise of this model as a tool for targeted therapeutic discovery in the most severely affected cell lineage from clinical patients with MFS.

## Materials and methods

### MFS patient selection

The Stanford Institutional Review Board (IRB) approved experiments involving human specimens. All patients included in this study gave informed consent for tissue banking and participation in human subject studies during elective cardiac surgery cases. Blanket research consent was obtained from surrogate decision makers for all included organ donor controls by the referring organ procurement organization. Blood samples used for iPSC derivation were collected under informed consent and based on the Stanford University Institutional Board Review approved protocol ID 23395.

*iTRAQ-1:* Two female MFS patients were selected to represent the varying clinical severity of aortic disease in individuals with distinct *FBN1* mutations. The “mild” aortic case had a genetic point-mutation located in the *N*-terminal-third of the *FBN1* gene, producing an Ile/Thr substitution at position 5726 (exon 46) in the epidermal growth factor (EGF)-like domain (*FBN1*: c.5726T > C, p.Ile1909Thr). This patient required surgical repair for severe mitral valve disease and underwent concurrent repair of a small (4.5 cm) aortic root aneurysm at age 27. In contrast, the “severe” case resulted from a 2 bp deletion at the junction between exons 46/47 producing a frameshift mutation and premature stop codon downstream of the mutation site (*FBN1*: c.6783-6784delGT). This patient underwent elective repair of a 5.0 cm aortic root aneurysm at age 20 and subsequently developed a type B aortic dissection, ultimately requiring repair of her thoracoabdominal aortic aneurysm. As an age- and sex-matched control, PBMC-derived iPSC SMCs from a healthy female age 24 were utilized (donated by Dr. Thomas Quertermous, Stanford Cardiovascular Institute, Stanford University).

*iTRAQ-2:* To ensure biologic and technical reproducibility, a second cohort included two male MFS patients with severe disease. The first patient had a c.493 C > T nonsense mutation in exon 5 with predicted nonsense-mediated decay and functional haploinsufficiency. This patient underwent aortic root replacement surgery at age 16, developed a type B aortic dissection requiring open repair of the descending thoracic aorta at age 27, and required aortic arch surgery at age 34. The second patient developed a 4.9 cm aortic root aneurysm at age 18 and underwent prophylactic aortic root replacement surgery. The original donor control line from iTRAQ-1 was used as a loading control and a second age- and sex-matched healthy male age 25 was added (normal donor cell line, donated by Dr. Joseph Wu, Stanford Cardiovascular Institute, Stanford University).

### Cell cultures

(a) *iPSC culture*: Human iPSC lines were produced at Stanford University Department of Genetics/Stem Cell Core Facilities. Briefly, cells were derived from PBMCs using co-infection with Sendai virus vectors encoding either KLF4, Sox2, Oct4 and Myc transcription factors, following Fusaki protocols for PBMC^57^. Cell lines were expanded on HESC-Matrigel (Corning, Tewkbury, MA) and cultivated first on mTeSR1 medium (StemCell Technologies, Vancouver, Canada) then adapted to mTeSR8-Plus medium (StemCell Technologies) and passaged using 0.5 mM EDTA. The medium was changed daily starting within the initial 3 days of culture. Reprogramming efficiency was assessed through TRA1–60 and SSEA4 immunostaining (Fig. 1B) and Sendai virus clearance was evaluated between passages 15–20 by RT PCR with Sendai specific primers.

(b) *iPSC differentiation (NC or LM progenitors subsequently differentiated into vascular SMCs).* Differentiation protocols have been adapted from previously published methodologies^9–11,23^. Briefly, chemically defined medium was used as a vehicle for differentiation factors [CDM (500 mL): 250 mL of IMDM (Gibco/Thermo Fisher Scientific, Sunnyvale, CA) + 250 mL F12 nutrient mix (Gibco/Thermo Fisher Scientific) + 5 mL chemically defined lipid concentrate (Sigma-Aldrich, Saint Louis, MO) + 250 µL of transferrin (R&D Systems, Minneapolis, MN) + 350 µL insulin (Roche, San Francisco, CA) + 20 µL of monothioglycerol (Sigma-Aldrich)]. For the NC differentiation, cells were grown in a CDM formula + SB431542 (10 μM, Sigma-Aldrich) + FGF2 (12 ng/mL, R&D Systems) for 7 days. For the early mesoderm formation, cells were initially grown in CDM + FGF2 (20 ng/mL, R&D Systems/ Fisher Scientific) + LY294002 (10 μM, Sigma-Aldrich) + BMP4 (10 ng/mL, R&D Systems/ Fisher Scientific) for 36 h. Further specification into LM required CDM + FGF2 (20 ng/mL, R&D Systems Fisher Scientific) + BMP4 (50 ng/mL, R&D Systems/Fischer Scientific) for another 3.5 days. Upon obtaining the intermediate populations, cell monolayers were dissociated with TrypLE (Gibco/Thermo Fisher) and cultured in SMC differentiation medium CDM + PDGF-BB (10 ng/mL, PeproTech, Rocky Hill, NJ) + TGF-β1 (2 ng/mL, PeproTech) for at least 12 days.

(c) *EB formation*. An AggreWell-400 kit was used to determine the capacity of the iPSC lines to form EB/spherical bodies. This assay was performed according to manufacturer protocol (StemCell Technologies). Cultures were first strained to separate EBs from unincorporated cells. Next, EBs/spheroids were counted under 10 × magnitude on a standard inverted microscope to determine the actual yield. Briefly, using a pipette, 50 μL of the EB/spheroid suspension was distributed into a flat-bottom 96-well plate, and spherical bodies were counted. The number of EB or spheroid yield was calculated as follows: Total number of EBs = EB/spheroid count (in 50 µL)/50 µL x Volume of EB/spheroid suspension (µL). The expected yield was approximately 1200 EBs/spheroids for 1 well of a 24-well plate which was very close to the normal standards. Spherical bodies were examined and micro-photographed 24 h post-culture when they were also replenished with serum free medium to induce spontaneous differentiation for another 48 h. At day 3 (72 h post EB formation onset) cells were tested by RT-PCR for specific markers of the three embryo layers, i) Ectoderm (SOX-1: 5′-TCCCCCGCGTGAACTG-3′, 5′-TTGAGGGCATTCTCTTGAGG-3′); ii) Mesoderm (Brachyury: 5″- TGCTTCCCTGAGACCCAGTT-3′, 5′-GATCACTTCTTTCCTTTGCATCAAG-3′); Ectoderm (SOX17: 5′-CGCACGGAATTTGAACAGTA-3′, 5′- GGATCAGGGACCTGTCACAC-3′).

(d) *FBN1 gene* mutations were identified by DNA sequencing. Karyotype stability tests based on Giemsa staining (G-Banding) of metaphase-chromosomes were performed on each of the iPSC cell lines. Specimens were internally examined by the Stanford University clinical Genetics Departments.

### 8-Plex iTRAQ analysis protein extraction

iPSC-derived SMCs were washed with 1 × PBS, manually dispersed and pelleted by centrifugation then snap frozen without protease or phosphatase inhibitors. Each frozen sample was then suspended in lysis buffer consisting of 8 M urea (U5378; Sigma-Aldrich), 100 mM Tris–HCl (pH 8.0), 10 mM dithiothreitol (DTT, Sigma-Aldrich). The digest was centrifuged at 10,000 × g for 30 min at 4 °C, and the supernatant was collected. The concentration of the supernatant was measured and divided into technical replicates. Next, to 200 μg protein 5 µL of 1 M DTT was added at 37 °C for 1 h and alkylated with 20 μL 1 M iodoacetamide at room temperature in the dark. Trypsin digestion (protein/trypsin ratio of 50:1) was performed for more than 12 h at 37 °C. Protein concentration was measured using the Bio-Rad protein assay kit (Bio-Rad, San Francisco, CA). For each run, 20 µg protein was taken and the buffer was replaced with 0.5 M triethylammonium bicarbonate, pH 8.5, followed by reduction, alkylation, trypsin digestion, iTRAQ labeling, and sample clean-up according to the manufacturer’s instructions (AB SCIEX).

### LC–MS/MS analysis

NanoLC was carried out using a Fisher Scientific NCS-3500RS nano pro-flow nano-cap-system. Tryptic peptides were loaded into a Precolumn Cartridge and separated on an acetonitrile gradient (ranging from 5 to 90%) on a C18 Nano LC column. Fractions were collected at 20-s intervals followed by Mass Spectrometry analysis on AB SCIEX TOF/TOF 5800 System (AB SCIEX). Mass spectra were acquired in reflectron positive ion mode. TOF/TOF tandem MS/MS fragmentation spectra were acquired for each ion, averaging 4000 laser shots per fragmentation spectrum on (excluding trypsin autolytic peptides and other known background ions) (performed by Applied Biomics, Freemont, CA).

### Database search, protein identification, and quantification

Proteins were digested into peptides by trypsin (into proteolytic/tryptic peptides). The resulted raw MS/MS data were submitted to MASCOT search engine (version 2.3, Matrix Science, London, UK) to search the SwissProt database. Database searching was restricted to tryptic peptides. Database searches were performed (a) without constraining protein molecular weight or isoelectric point; (b) software default tolerance of variable methylthiolation of cysteine, oxidation of methionine residues, fixed N-terminal- and lysine-modifications with iTRAQ labels; and (c) with one missed cleavage allowed in the search parameters. Peptide sequences with either protein score confidence interval (C.I.) % or Ion C.I. % greater than 95 were accepted as matches with database sequences. Protein identifiers were converted to official Gene Name nomenclature using the UniProt ‘Retrive/ID Mapping’ tool (https://www.uniprot.org/uploadlists/). Protein ratios between LM and NC samples were then determined by calculating the geometric average of peptide ratios, permitting both unique and homologous peptide sequences in the analysis. Abundance ratio calculations included corrections for overlapping isotopic contributions.

### Western blot

Protein-extracts from iPSC-derived or aortic root/ascending aortic tissue lysates (MFS patients: n = 3; donor control: n = 1) were resolved by SDS-PAGE (10% A/BA Novex Bis/Tris gels, Thermo Fisher). Protein fractions separated onto the gels were transferred to PVDF membranes (Thermo Fisher). The membranes were blocked with SeaBlock solution (Thermo Fisher) for 60 min and then probed with primary antibodies to detect: MRC2 (Abcam, Cambridge, MA), MMP2 (Cell Signaling Technology, Boston, MA), Col 1A1 (Abcam), Integrin αV (Abcam), and GAPDH (Cell Signaling Technology) as loading control/house-keeping marker. Secondary anti-rabbit-HRP (Sigma-Aldrich) and anti-mouse-HRP (Sigma-Aldrich) antibodies were used to detect primary immuno-complexes. Detection was performed using the ECL system (Thermo Fisher).

### Immunocytochemistry/immunofluorescence (ICC/IF):

iPSC-derived LM and NC progenitors or differentiated SMCs were plated on glass coverslips coated with Matrigel (Corning) and 24 h later fixed with ice-cold 90% ethanol. Fixed cells were rehydrated with 1 × PBS, permeabilized with 0.2% Triton solution in 1 × PBS and non-specific binding diminished using Sea Block Blocking Solution (Thermo Scientific). Cells were incubated with primary antibodies (all purchased from Abcam, Burlingame, CA) targeting the following markers: (i) myocyte enhancer factor-2c (MEF2c) and homeobox protein NKX2.5 (LM progenitor cells); (ii) Nestin (NC progenitor cells); (iii) Calponin 1 (CNN1) and Transgelin (TGLN) (differentiated SMCs); or (iv) immunoglobulin isotype control, then stained with the appropriate Alexa Fluor secondary antibodies (Thermo Fisher Scientific). Preps were counter-stained for chromatin with Hoechst reagent (1 μg/mL) (Sigma-Aldrich). Fluorescent images were captured using a Leica DM5500B Upright Microscope and Photometrics CoolSNAP HQ2 CCD camera, using HC Plan Apo 25-mm objectives.

### Matrix metalloproteinase-2 (MMP2) zymography analysis

SMC culture supernatants were collected and protein concentrations were determined by bicinchoninic acid assay (BCA) according to manufacturer’s instructions (Thermo Scientific/Pierce, Rockford, IL). 10 μg protein was resolved by non-denaturing electrophoresis through a 10% gelatin gel (Invitrogen, Carlsbad, CA). The molecular sizes of gelatinolytic activities were determined using 10 µl of MMP marker (Cosmobio, Tokyo, Japan). Quantification by densitometry was performed using ImageJ software (National Institutes of Health, Bethesda, MD) on inverted images of gel.

### Cell adhesion assay

Following manufacturers protocol for CytoSelect Cell Adhesion Assay kit (Cell Biolabs, San Diego, CA), iPSC-derived SMCs were gently treated with Trypsin solution TrypleX (Gibco/Thermo Fisher) for 3 min. Detached cells were washed with 1xPBS and pelted by centrifugation at 200 g. Cells were re-suspended in normal culture medium and plated in a 48-well cell culture plate where groups of 8 wells were pre-coated by manufacturer with either: Collagen II or IV, Fibronectin, Laminin, Fibrinogen or BSA (as a negative control for adhesion). After 1 h incubation at 37 °C/5.2% CO_2_, non-adherent cells were removed by washing three times with serum-free DMEM, while adherent cells were fixed and stain with Crystal blue dye solution for 20 min. After the dye was discarded, cells were washed with 1xPBS and the dye extracted with an acetic acid solution. All reagents were provided by the kit. Dye confrontations retained by the cells were measured by using a plate reader and a λ = 540 nm cut-off filter. Adhesion was evaluated by the amount of dye retention.

### Enzyme-linked immunosorbent assay (ELISA)

Protein was extracted from aortic root and ascending aortic tissue (MFS patients: n = 6; donor control patients: n = 4) and the protein concentration determined by Bio-Rad BCA method (Bio-Rad, San Francisco, CA). Human MRC2 ELISA kit (Cat#EKE61298, Cambridge, ON, Canada) and Human CD51 (Integrin αV) ELISA Kit (Cat#ab235650, Abcam) were used per each manufacturer protocol. To measure total integrin αV levels, 10 μg total protein was used. To measure MRC2 levels, 20 μg total protein was utilized. Results were expressed as concentration of each marker (pg/mL) based on standard regression curb, per each manufacturer protocol. All samples were measured in triplicate × 2.

### Statistical analysis

All data are presented as means ± SD of 2 independent experiments unless otherwise stated. Statistical differences between 2 groups were determined by non-parametric analysis, by Mann–Whitney test (GraphPad Prism 7, San Diego, CA). A *P-*value of < 0.05 was considered statistically significant. Significance was assigned based on the *P*-value (*P < 0.05, **P < 0.01, ***P < 0.001). All experiments have been performed with technical replicates or repeats.

## Supplementary information

Supplementary Information 1. **Table 1**. Comprehensive proteomic profile of LM and NC SMCs (MFS vs. donor control). Table includes the following categories: (i) abundance expressed as fold change compared to the reference control); (ii) technical parameters regarding peptide analysis; and (iii) full name and coding of each protein. **Table 2**. List of proteins with 1.2-old increase or decrease compared to reference control.

Supplementary Information 2. **Supplementary Fig. 1**. Western blotting technical conditions. (**A**) Human iPSC SMC; (**B**) Human aortic tissue Data in support to Fig. 4.
