# Supplementary material for: Quantitative proteomics reveal lineage-specific protein profiles in iPSC-derived Marfan syndrome smooth muscle cells
Source: Sci Rep. 2020 Nov 23;10:20392. doi: 10.1038/s41598-020-77274-w (PMC7683538; doi:10.1038/s41598-020-77274-w)
Supplement: Supplementary file 1 — Supplementary Information 1. Table 1. Comprehensive proteomic profile of LM and NC SMCs (MFS vs. donor control). Table includes the following categories: (i) abundance expressed as fold change compared to the reference control); (ii) technical parameters regarding peptide analysis; and (iii) full name and coding of each protein. Table 2. List of proteins with 1.2-old increase or decrease compared to reference control. [file 41598_2020_77274_MOESM1_ESM.pptx]

## Slide 1
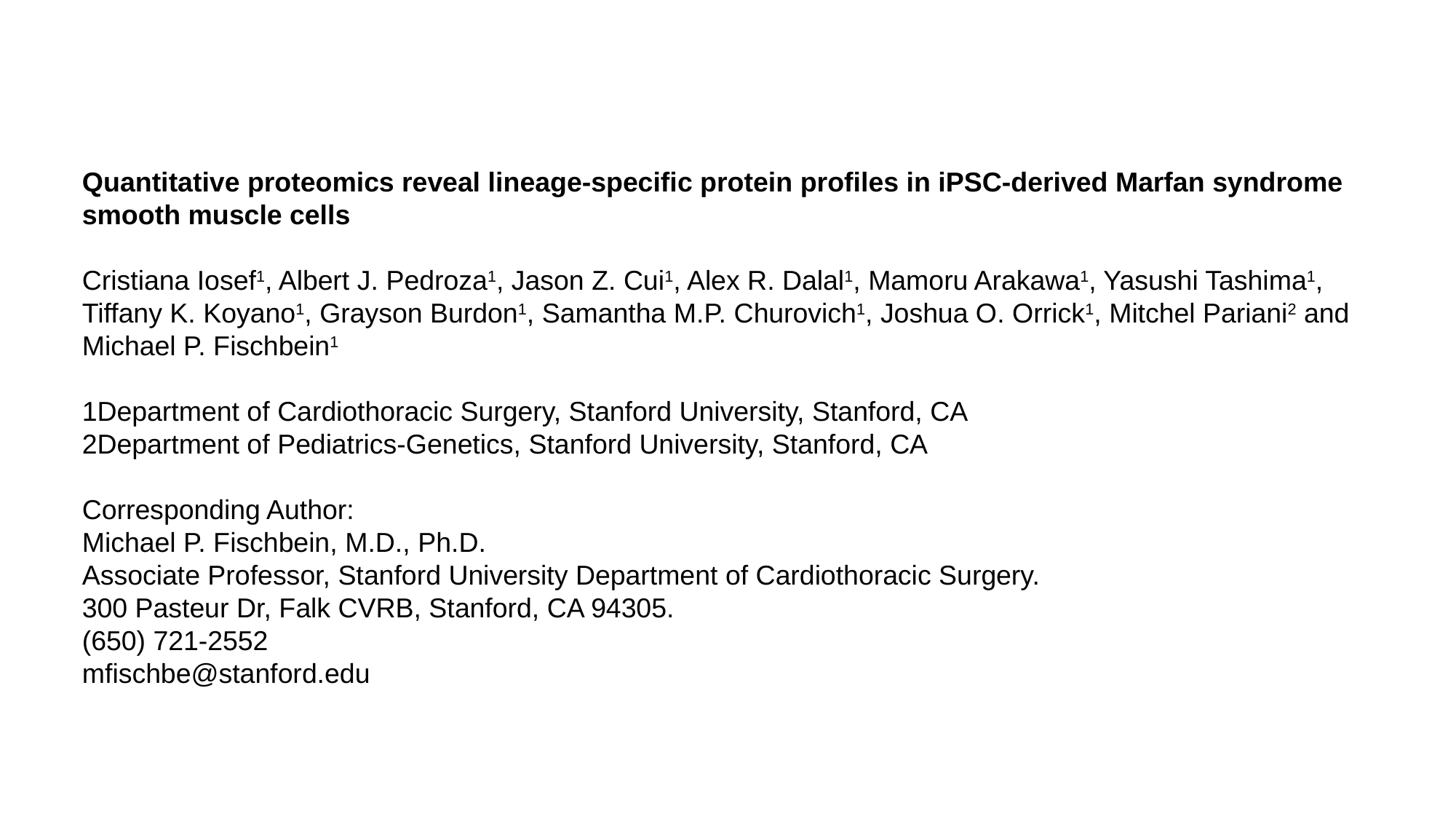

Quantitative proteomics reveal lineage-specific protein profiles in iPSC-derived Marfan syndrome smooth muscle cells
Cristiana Iosef1, Albert J. Pedroza1, Jason Z. Cui1, Alex R. Dalal1, Mamoru Arakawa1, Yasushi Tashima1, Tiffany K. Koyano1, Grayson Burdon1, Samantha M.P. Churovich1, Joshua O. Orrick1, Mitchel Pariani2 and Michael P. Fischbein1
1Department of Cardiothoracic Surgery, Stanford University, Stanford, CA
2Department of Pediatrics-Genetics, Stanford University, Stanford, CA
Corresponding Author:
Michael P. Fischbein, M.D., Ph.D.
Associate Professor, Stanford University Department of Cardiothoracic Surgery.
300 Pasteur Dr, Falk CVRB, Stanford, CA 94305.
(650) 721-2552
mfischbe@stanford.edu

## Slide 2
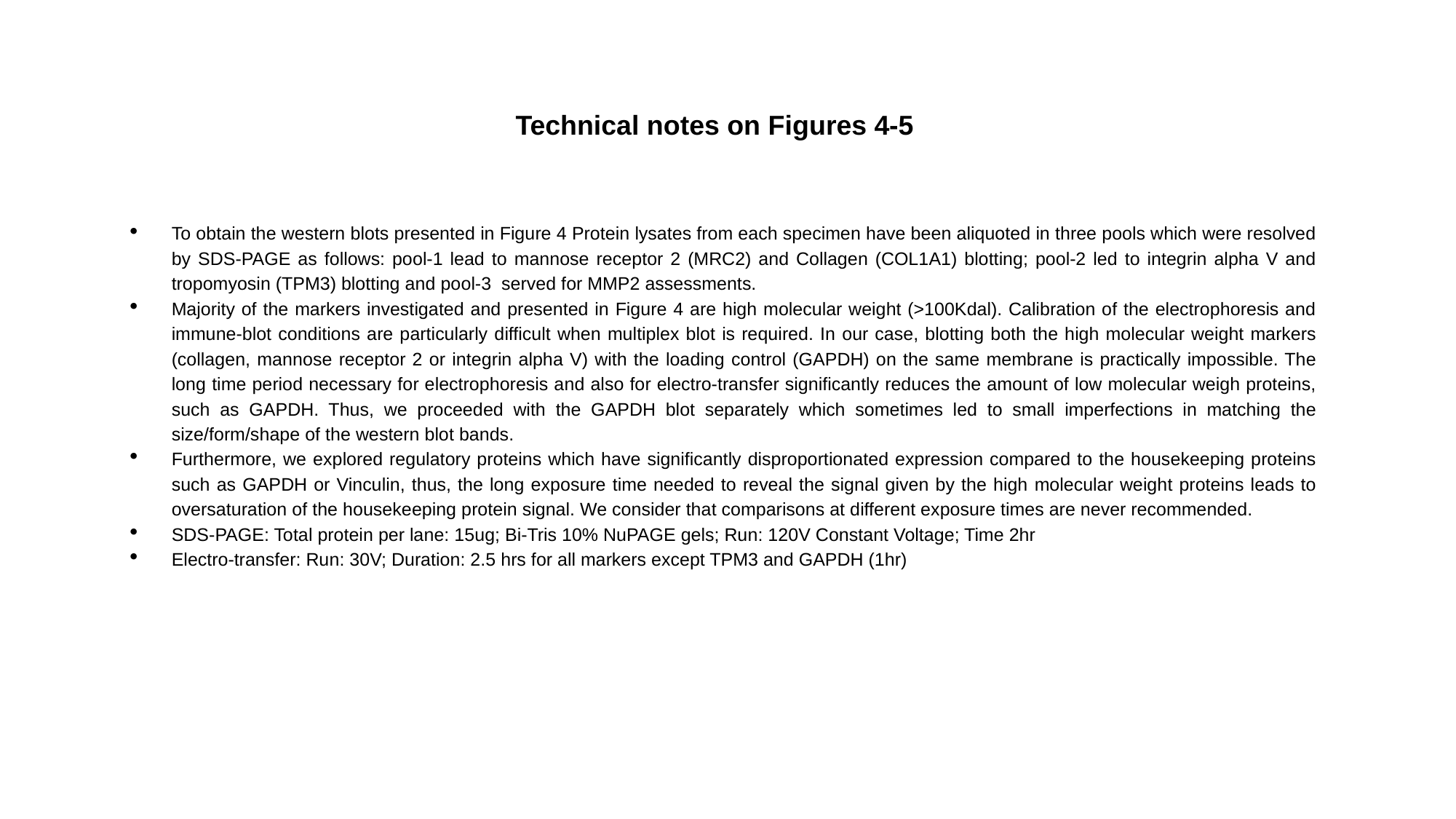

Technical notes on Figures 4-5
To obtain the western blots presented in Figure 4 Protein lysates from each specimen have been aliquoted in three pools which were resolved by SDS-PAGE as follows: pool-1 lead to mannose receptor 2 (MRC2) and Collagen (COL1A1) blotting; pool-2 led to integrin alpha V and tropomyosin (TPM3) blotting and pool-3 served for MMP2 assessments.
Majority of the markers investigated and presented in Figure 4 are high molecular weight (>100Kdal). Calibration of the electrophoresis and immune-blot conditions are particularly difficult when multiplex blot is required. In our case, blotting both the high molecular weight markers (collagen, mannose receptor 2 or integrin alpha V) with the loading control (GAPDH) on the same membrane is practically impossible. The long time period necessary for electrophoresis and also for electro-transfer significantly reduces the amount of low molecular weigh proteins, such as GAPDH. Thus, we proceeded with the GAPDH blot separately which sometimes led to small imperfections in matching the size/form/shape of the western blot bands.
Furthermore, we explored regulatory proteins which have significantly disproportionated expression compared to the housekeeping proteins such as GAPDH or Vinculin, thus, the long exposure time needed to reveal the signal given by the high molecular weight proteins leads to oversaturation of the housekeeping protein signal. We consider that comparisons at different exposure times are never recommended.
SDS-PAGE: Total protein per lane: 15ug; Bi-Tris 10% NuPAGE gels; Run: 120V Constant Voltage; Time 2hr
Electro-transfer: Run: 30V; Duration: 2.5 hrs for all markers except TPM3 and GAPDH (1hr)

## Slide 3
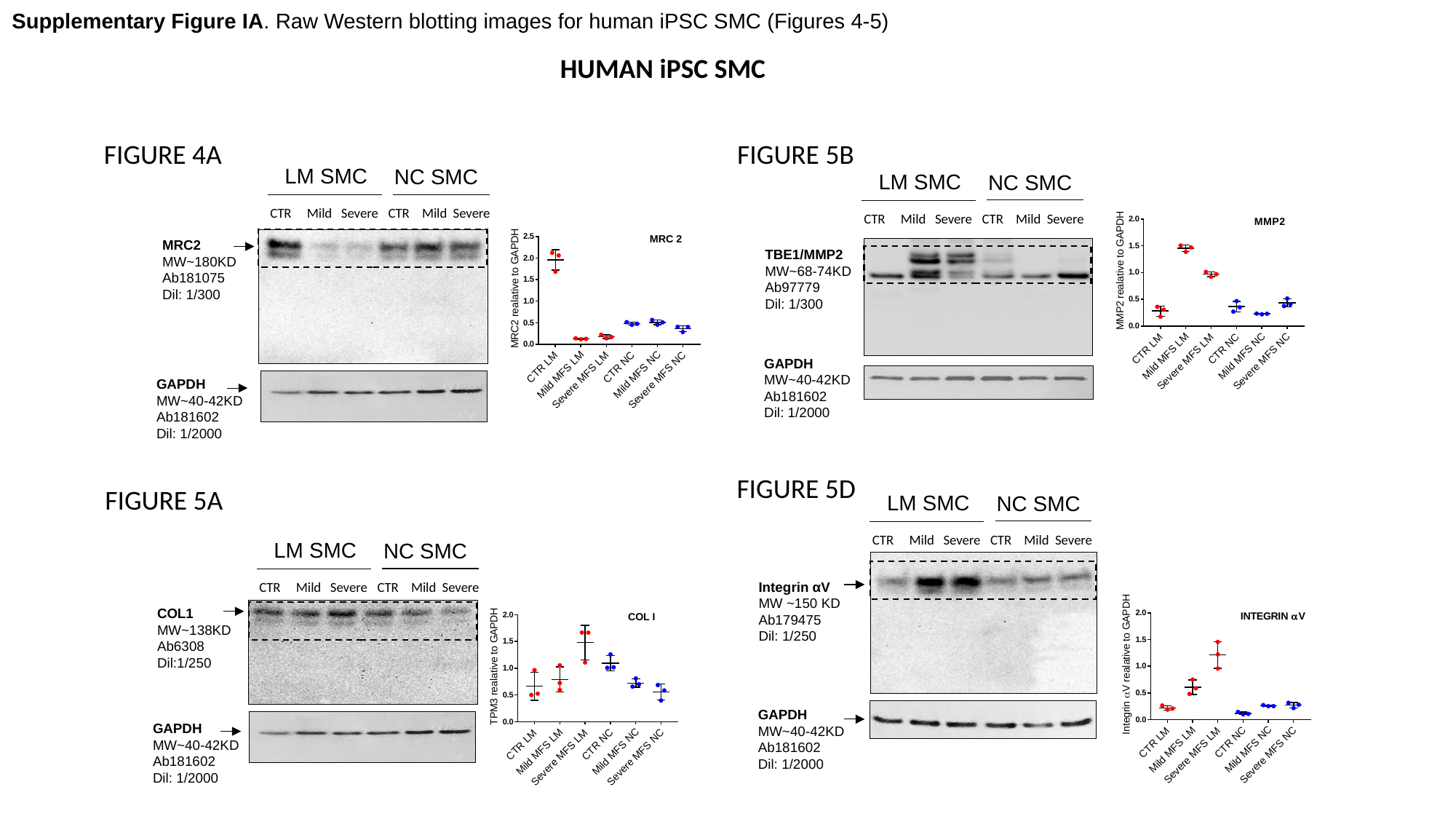

Supplementary Figure IA. Raw Western blotting images for human iPSC SMC (Figures 4-5)
HUMAN iPSC SMC
FIGURE 4A
FIGURE 5B
LM SMC
NC SMC
CTR Mild Severe
CTR Mild Severe
TBE1/MMP2
MW~68-74KD
Ab97779
Dil: 1/300
GAPDH
MW~40-42KD
Ab181602
Dil: 1/2000
LM SMC
NC SMC
CTR Mild Severe
CTR Mild Severe
MRC2
MW~180KD
Ab181075
Dil: 1/300
GAPDH
MW~40-42KD
Ab181602
Dil: 1/2000
FIGURE 5D
LM SMC
NC SMC
CTR Mild Severe
CTR Mild Severe
Integrin αV
MW ~150 KD
Ab179475
Dil: 1/250
GAPDH
MW~40-42KD
Ab181602
Dil: 1/2000
FIGURE 5A
LM SMC
NC SMC
CTR Mild Severe
CTR Mild Severe
COL1
MW~138KD
Ab6308
Dil:1/250
GAPDH
MW~40-42KD
Ab181602
Dil: 1/2000

## Slide 4
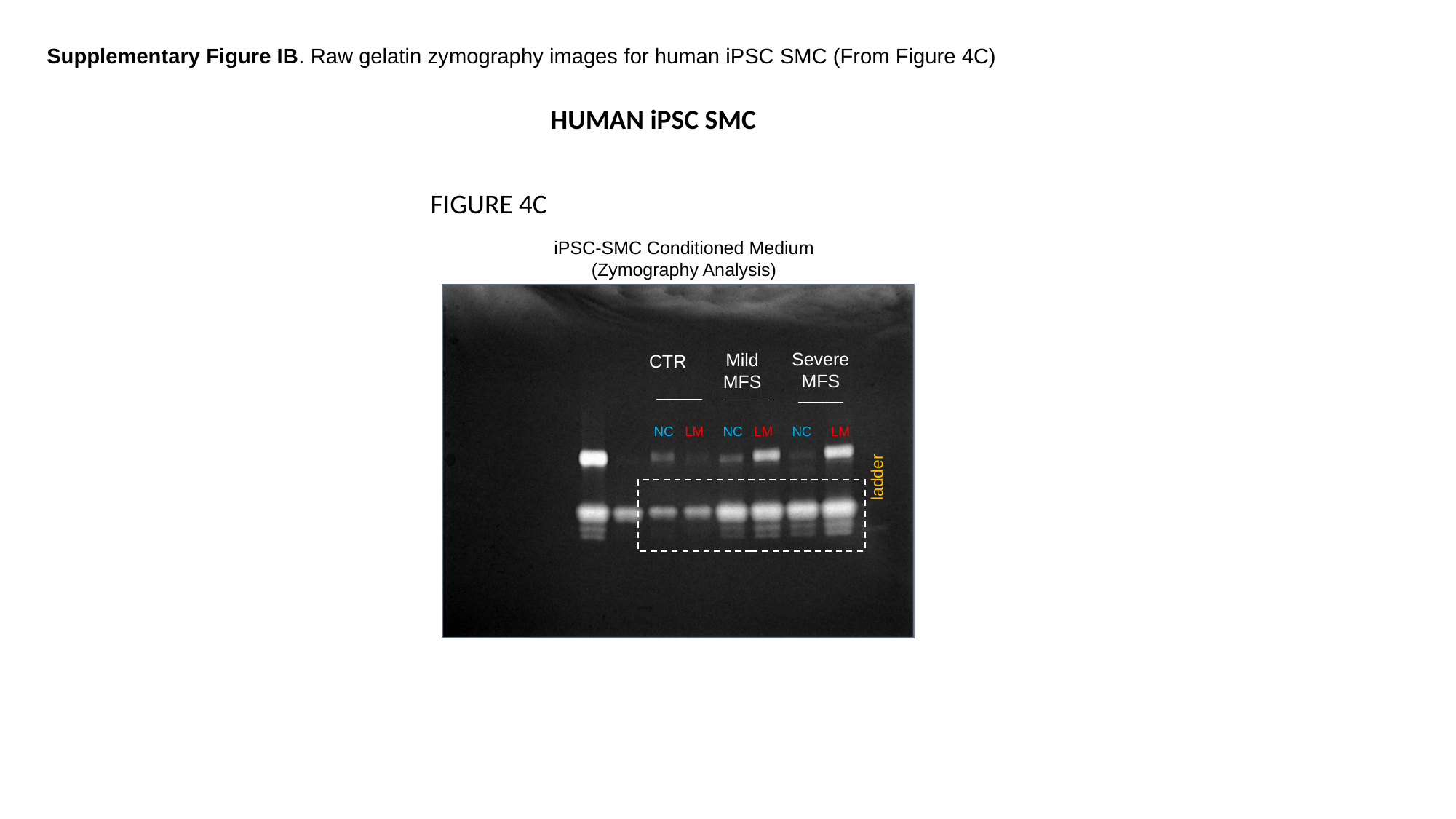

Supplementary Figure IB. Raw gelatin zymography images for human iPSC SMC (From Figure 4C)
HUMAN iPSC SMC
FIGURE 4C
iPSC-SMC Conditioned Medium
(Zymography Analysis)
Severe
MFS
Mild
MFS
CTR
 NC LM NC LM NC LM
ladder

## Slide 5
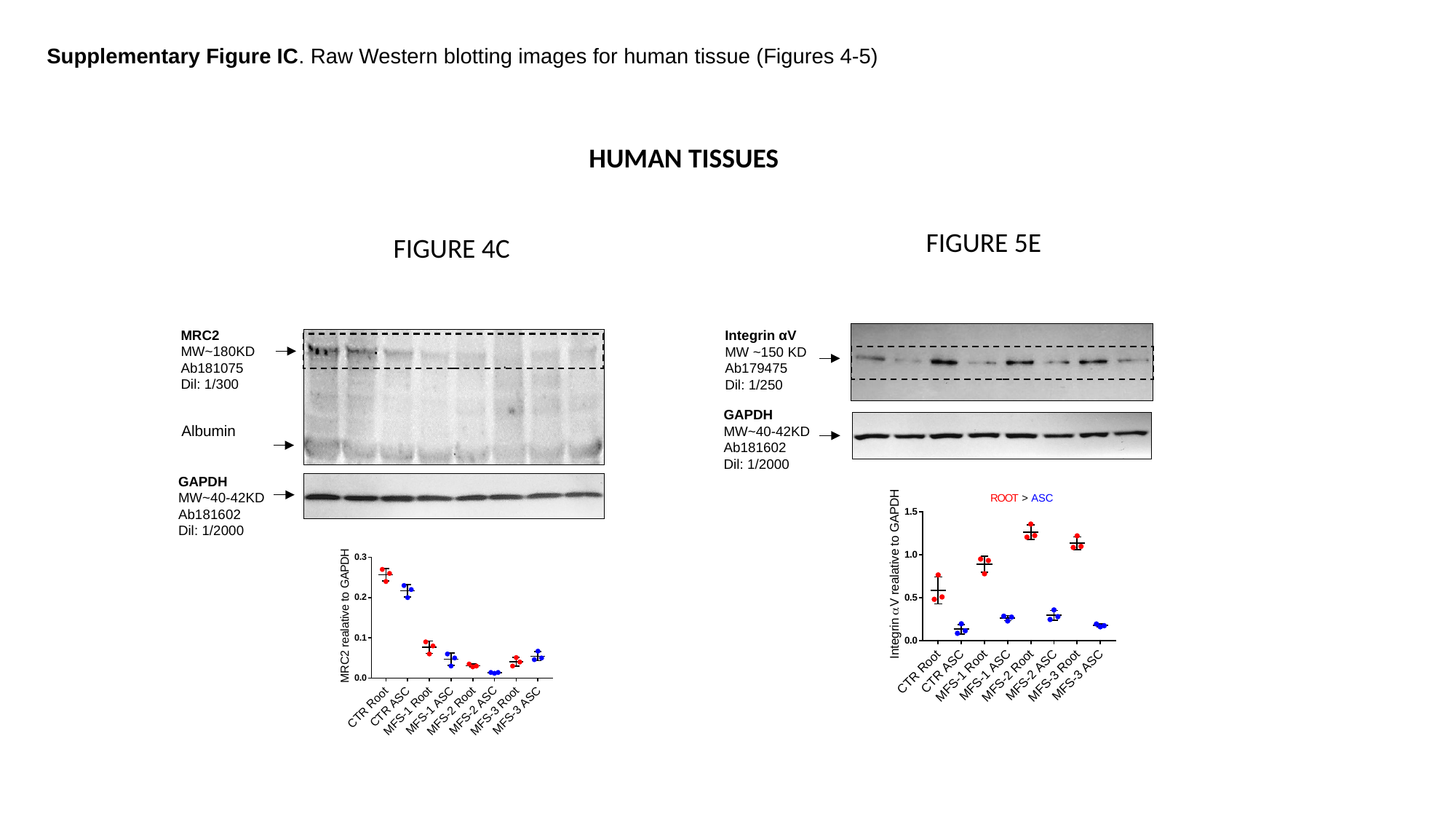

Supplementary Figure IC. Raw Western blotting images for human tissue (Figures 4-5)
HUMAN TISSUES
FIGURE 5E
FIGURE 4C
MRC2
MW~180KD
Ab181075
Dil: 1/300
Integrin αV
MW ~150 KD
Ab179475
Dil: 1/250
GAPDH
MW~40-42KD
Ab181602
Dil: 1/2000
Albumin
GAPDH
MW~40-42KD
Ab181602
Dil: 1/2000

## Slide 6
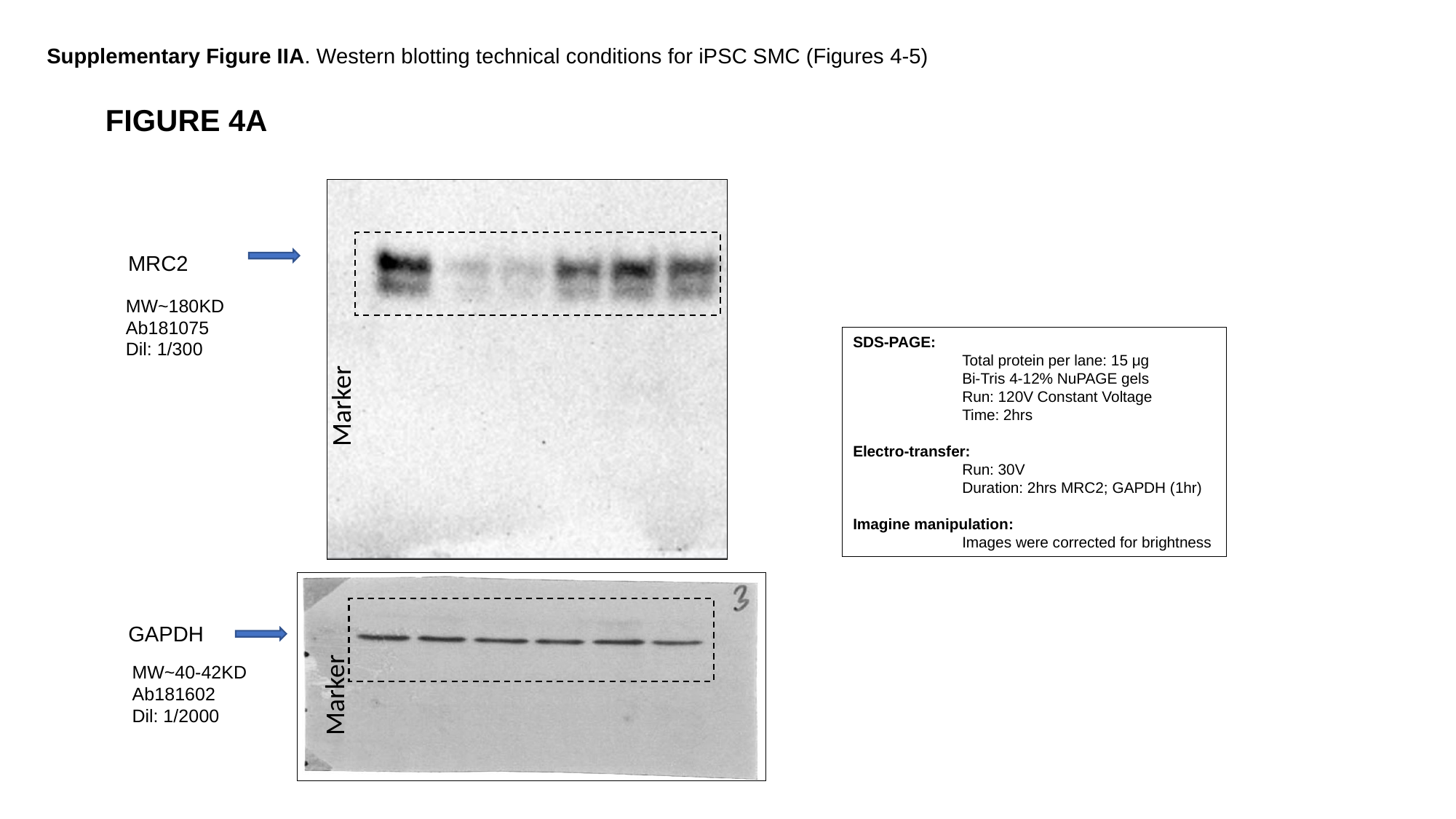

Supplementary Figure IIA. Western blotting technical conditions for iPSC SMC (Figures 4-5)
FIGURE 4A
MRC2
MW~180KD
Ab181075
Dil: 1/300
SDS-PAGE:
	Total protein per lane: 15 μg
	Bi-Tris 4-12% NuPAGE gels
	Run: 120V Constant Voltage
	Time: 2hrs
Electro-transfer:
	Run: 30V
	Duration: 2hrs MRC2; GAPDH (1hr)
Imagine manipulation:
	Images were corrected for brightness
Marker
GAPDH
MW~40-42KD
Ab181602
Dil: 1/2000
Marker

## Slide 7
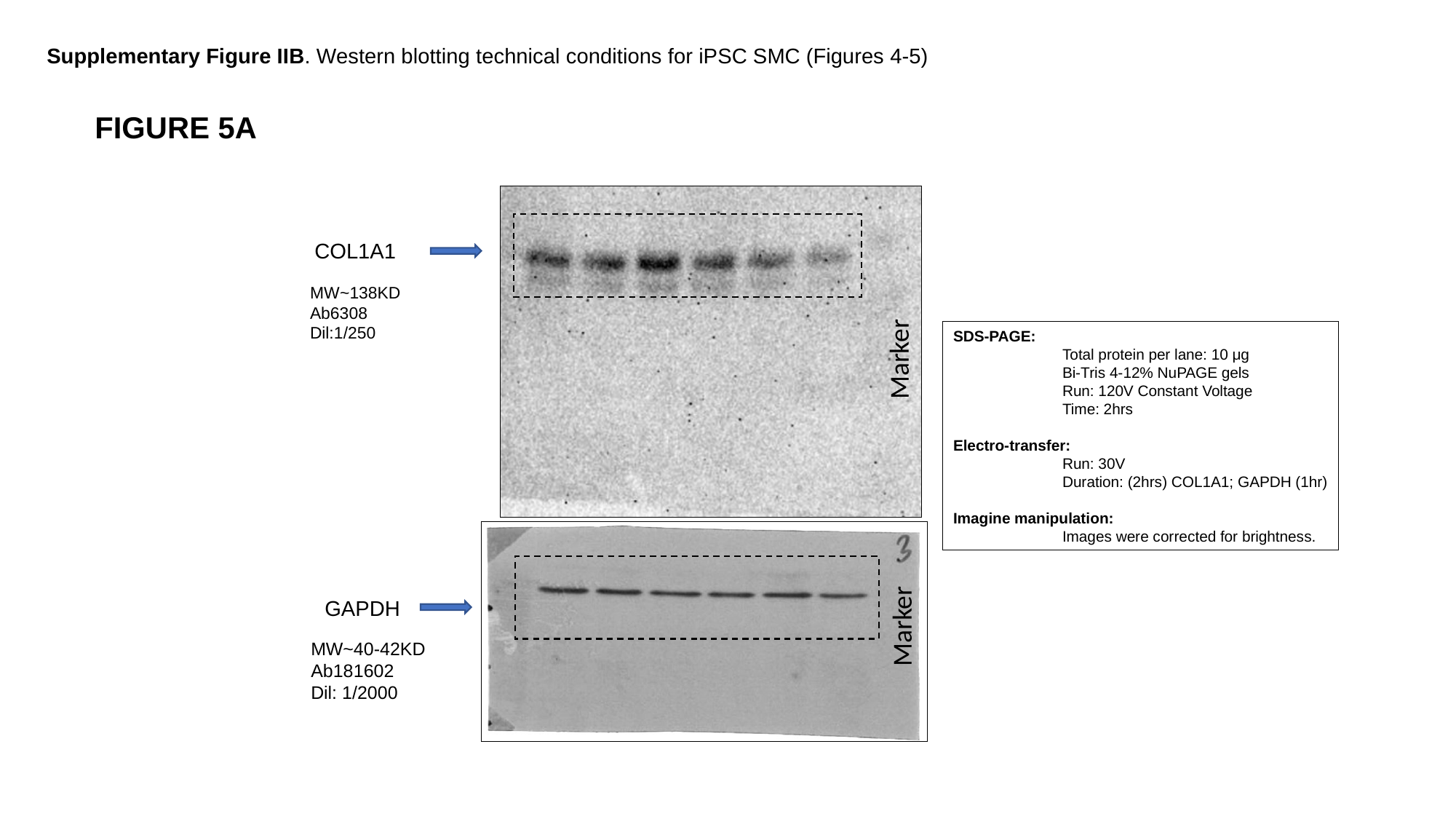

Supplementary Figure IIB. Western blotting technical conditions for iPSC SMC (Figures 4-5)
FIGURE 5A
COL1A1
MW~138KD
Ab6308
Dil:1/250
SDS-PAGE:
	Total protein per lane: 10 μg
	Bi-Tris 4-12% NuPAGE gels
	Run: 120V Constant Voltage
	Time: 2hrs
Electro-transfer:
	Run: 30V
	Duration: (2hrs) COL1A1; GAPDH (1hr)
Imagine manipulation:
	Images were corrected for brightness.
Marker
GAPDH
Marker
MW~40-42KD
Ab181602
Dil: 1/2000

## Slide 8
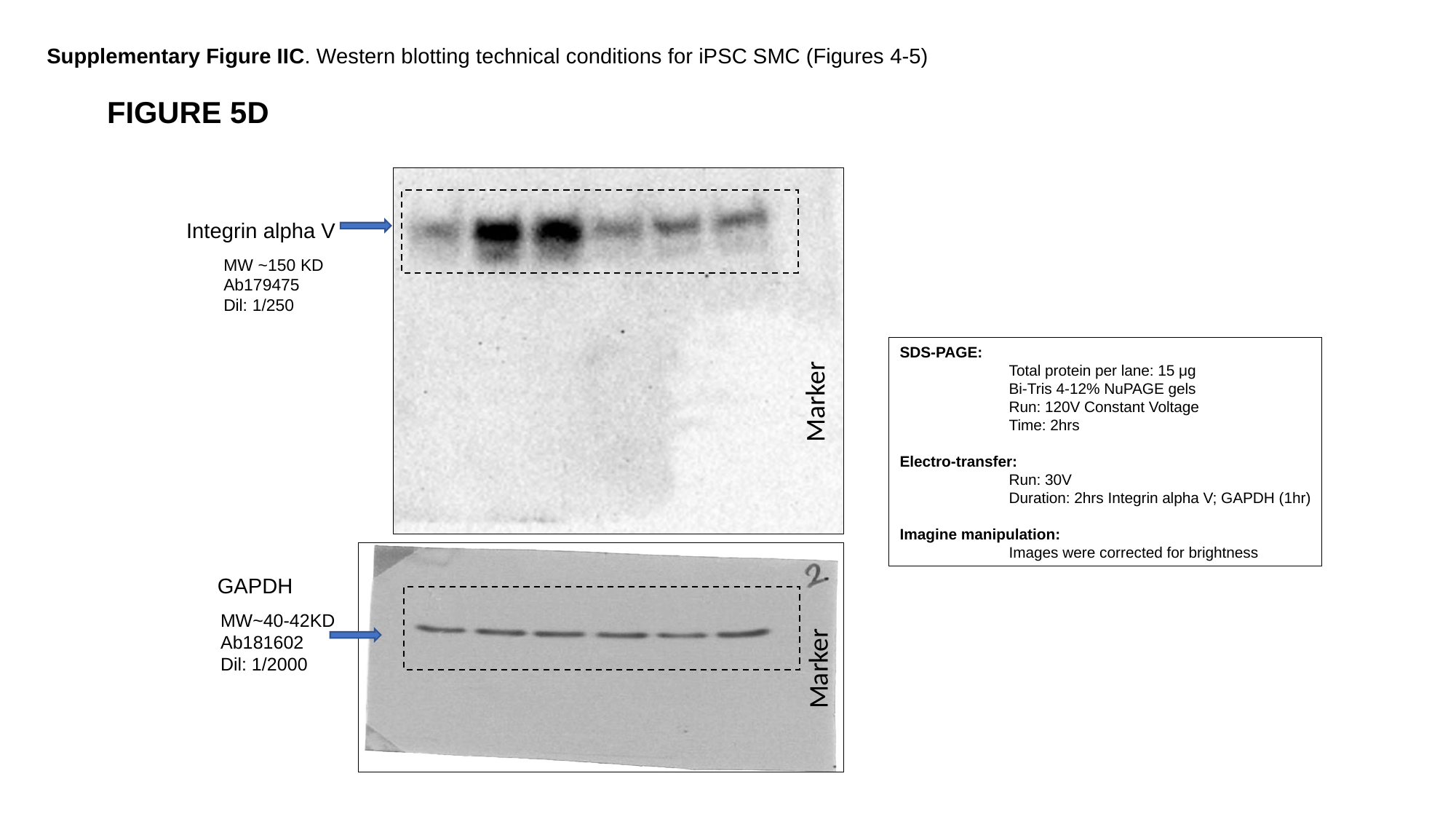

Supplementary Figure IIC. Western blotting technical conditions for iPSC SMC (Figures 4-5)
FIGURE 5D
Integrin alpha V
MW ~150 KD
Ab179475
Dil: 1/250
SDS-PAGE:
	Total protein per lane: 15 μg
	Bi-Tris 4-12% NuPAGE gels
	Run: 120V Constant Voltage
	Time: 2hrs
Electro-transfer:
	Run: 30V
	Duration: 2hrs Integrin alpha V; GAPDH (1hr)
Imagine manipulation:
	Images were corrected for brightness
Marker
GAPDH
MW~40-42KD
Ab181602
Dil: 1/2000
Marker

## Slide 9
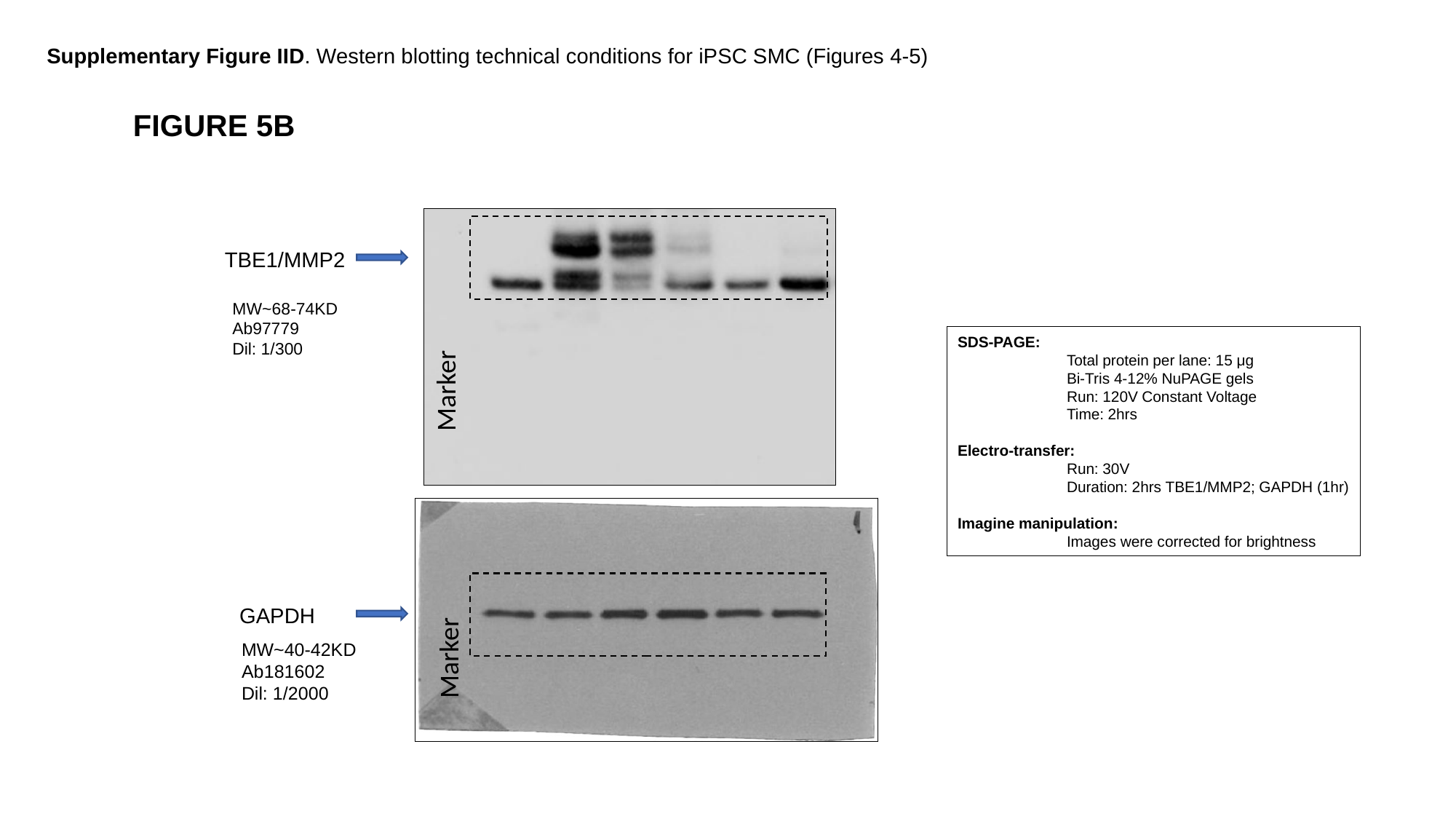

Supplementary Figure IID. Western blotting technical conditions for iPSC SMC (Figures 4-5)
FIGURE 5B
TBE1/MMP2
MW~68-74KD
Ab97779
Dil: 1/300
SDS-PAGE:
	Total protein per lane: 15 μg
	Bi-Tris 4-12% NuPAGE gels
	Run: 120V Constant Voltage
	Time: 2hrs
Electro-transfer:
	Run: 30V
	Duration: 2hrs TBE1/MMP2; GAPDH (1hr)
Imagine manipulation:
	Images were corrected for brightness
Marker
GAPDH
MW~40-42KD
Ab181602
Dil: 1/2000
Marker

## Slide 10
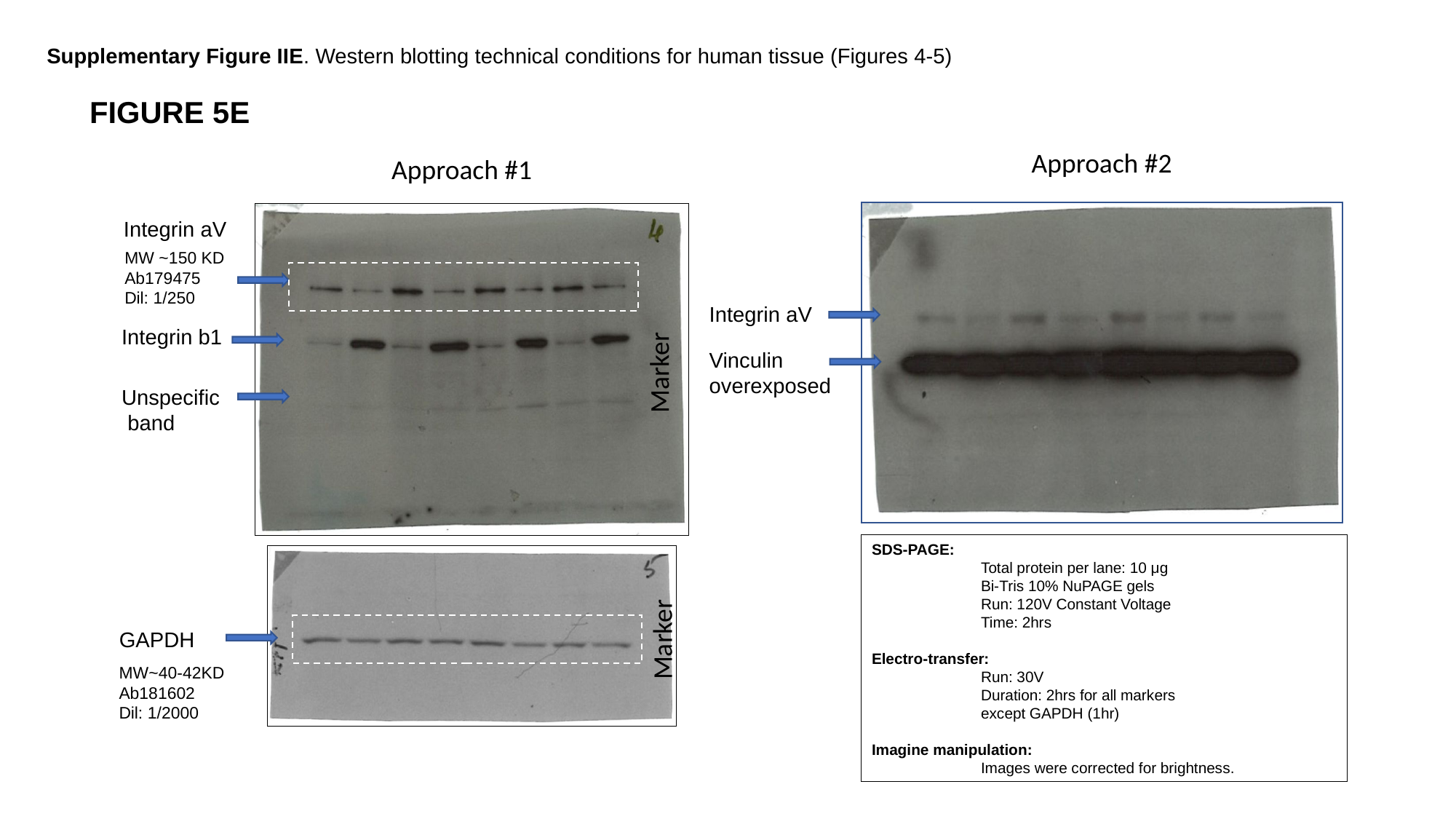

Supplementary Figure IIE. Western blotting technical conditions for human tissue (Figures 4-5)
FIGURE 5E
Approach #2
Approach #1
Integrin aV
MW ~150 KD
Ab179475
Dil: 1/250
Integrin aV
Integrin b1
Vinculin
overexposed
Marker
Unspecific
 band
SDS-PAGE:
	Total protein per lane: 10 μg
	Bi-Tris 10% NuPAGE gels
	Run: 120V Constant Voltage
	Time: 2hrs
Electro-transfer:
	Run: 30V
	Duration: 2hrs for all markers
	except GAPDH (1hr)
Imagine manipulation:
	Images were corrected for brightness.
Marker
GAPDH
MW~40-42KD
Ab181602
Dil: 1/2000

## Slide 11
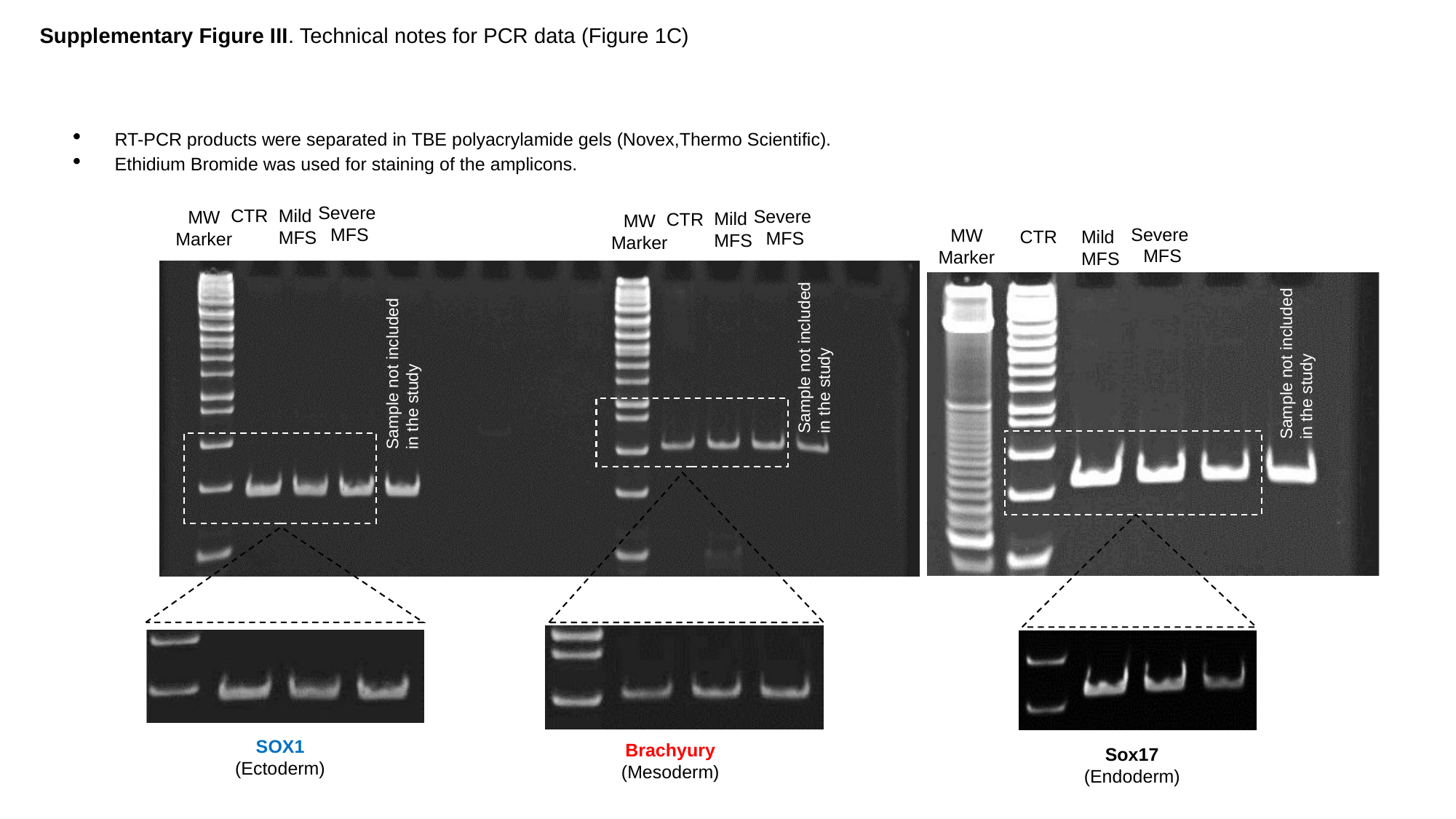

Supplementary Figure III. Technical notes for PCR data (Figure 1C)
RT-PCR products were separated in TBE polyacrylamide gels (Novex,Thermo Scientific).
Ethidium Bromide was used for staining of the amplicons.
Severe
 MFS
Mild
 MFS
CTR
Severe
 MFS
MW
Marker
Mild
 MFS
CTR
MW
Marker
Severe
 MFS
MW
Marker
CTR
Mild
 MFS
Sample not included
in the study
Sample not included
in the study
Sample not included
in the study
SOX1
(Ectoderm)
Brachyury
(Mesoderm)
Sox17
(Endoderm)
